# Periodontal disease–related nonalcoholic fatty liver disease and nonalcoholic steatohepatitis: An emerging concept of oral‐liver axis

**DOI:** 10.1111/prd.12387

**Published:** 2021-08-31

**Authors:** Ryutaro Kuraji, Satoshi Sekino, Yvonne Kapila, Yukihiro Numabe

**Affiliations:** ^1^ Department of Life Science Dentistry The Nippon Dental University Tokyo Japan; ^2^ Department of Periodontology The Nippon Dental University School of Life Dentistry at Tokyo Tokyo Japan; ^3^ Department of Orofacial Sciences University of California San Francisco School of Dentistry San Francisco California USA

**Keywords:** gut microbial dysbiosis, metabolic syndrome, NAFLD, NASH, obesity, periodontal disease, periodontopathic bacteria, systemic inflammation

## Abstract

Periodontal disease, a chronic inflammatory disease of the periodontal tissues, is not only a major cause of tooth loss, but it is also known to exacerbate/be associated with various metabolic disorders, such as obesity, diabetes, dyslipidemia, and cardiovascular disease. Recently, growing evidence has suggested that periodontal disease has adverse effects on the pathophysiology of liver disease. In particular, nonalcoholic fatty liver disease, a hepatic manifestation of metabolic syndrome, has been associated with periodontal disease. Nonalcoholic fatty liver disease is characterized by hepatic fat deposition in the absence of a habitual drinking history, viral infections, or autoimmune diseases. A subset of nonalcoholic fatty liver diseases can develop into more severe and progressive forms, namely nonalcoholic steatohepatitis. The latter can lead to cirrhosis and hepatocellular carcinoma, which are end‐stage liver diseases. Extensive research has provided plausible mechanisms to explain how periodontal disease can negatively affect nonalcoholic fatty liver disease and nonalcoholic steatohepatitis, namely via hematogenous or enteral routes. During periodontitis, the liver is under constant exposure to various pathogenic factors that diffuse systemically from the oral cavity, such as bacteria and their by‐products, inflammatory cytokines, and reactive oxygen species, and these can be involved in disease promotion of nonalcoholic fatty liver disease and nonalcoholic steatohepatitis. Also, gut microbiome dysbiosis induced by enteral translocation of periodontopathic bacteria may impair gut wall barrier function and promote the transfer of hepatotoxins and enterobacteria to the liver through the enterohepatic circulation. Moreover, in a population with metabolic syndrome, the interaction between periodontitis and systemic conditions related to insulin resistance further strengthens the association with nonalcoholic fatty liver disease. However, most of the pathologic links between periodontitis and nonalcoholic fatty liver disease in humans are provided by epidemiologic observational studies, with the causal relationship not yet being established. Several systematic and meta‐analysis studies also show conflicting results. In addition, the effect of periodontal treatment on nonalcoholic fatty liver disease has hardly been studied. Despite these limitations, the global burden of periodontal disease combined with the recent nonalcoholic fatty liver disease epidemic has important clinical and public health implications. Emerging evidence suggests an association between periodontal disease and liver diseases, and thus we propose the term periodontal disease–related nonalcoholic fatty liver disease or periodontal disease–related nonalcoholic steatohepatitis. Continued efforts in this area will pave the way for new diagnostic and therapeutic approaches based on a periodontologic viewpoint to address this life‐threatening liver disease.

## 1 BACKGROUND

Periodontal disease is a common chronic inflammatory and infectious disease that is caused by an oral biofilm–mediated microbial dysbiosis that is predominantly comprised of anaerobic gram‐negative bacteria, namely periodontopathic bacteria.^1^, ^2^ These biofilms are a continually renewing storehouse of lipopolysaccharide and other microbial molecules that are derived from the resident gram‐negative bacteria. Biofilm components have ready access to the periodontal tissues and host circulation. Microbial challenges also initiate and perpetuate host immune responses in the periodontal tissues, resulting in production of high levels of inflammatory mediators and tissue‐destructive enzymes. These responses, in turn, lead to periodontal tissue destruction and tooth loss.^3^ The products from inflamed periodontal tissues also enter the circulation and enhance susceptibility to systemic diseases via several pathways.^1^

In the field of research related to periodontal medicine, few papers to date have addressed the relationship between periodontal disease and the organs of the digestive system. Meanwhile, the relationship between periodontal disease and liver disease has received growing attention in recent years. The liver is the largest organ in the digestive system, and it plays an important role in maintaining the health of living organisms.^4^ During the process of digestion, nutrients in food are absorbed through the numerous fine capillaries of the intestinal wall and they are carried into the veins.^5^ These veins merge into larger veins and ultimately enter the liver through the portal vein. The liver removes bacteria and other foreign matter from the blood that enters through the portal vein, and it further breaks down many nutrients that have been absorbed by the intestine.^4^ Blood rich in nutrients then recirculates for use throughout the body.

Liver diseases occur due to various causes, including infectious diseases, pharmaceutical use, toxins, ischemia, and autoimmune diseases. Many liver diseases cause liver cell damage, necrosis, and subsequent development of hepatic dysfunction, which leads to symptoms due to both the liver disease itself (eg, jaundice caused by acute hepatitis) and complications of the liver disease (eg, acute gastrointestinal bleeding as a result of liver cirrhosis and portal hypertension). Liver diseases such as hepatitis (which starts with a fatty liver caused by excessive alcohol consumption) and viral hepatitis are well known. However, in recent years, hepatitis and liver cirrhosis caused by fatty liver in the absence of alcohol consumption or in the presence of low alcohol consumption and without a viral infection have also been identified and are attracting attention.^6^, ^7^

In nonalcoholic fatty liver disease there is a fatty liver with hepatic fat deposits in the absence of habitual drinking, viral infections, or autoimmune diseases.^6^, ^7^ In particular, guidelines regarding the amount of ethanol consumed have been set at less than 30 g for men and less than 20 g for women for diagnosing nonalcoholic fatty liver disease. Nonalcoholic fatty liver disease is strongly associated with insulin resistance and metabolic syndrome because many cases of nonalcoholic fatty liver disease arise from conditions such as obesity, diabetes, dyslipidemia, and hypertension.^7^, ^8^, ^9^, ^10^ Nonalcoholic fatty liver disease has a high worldwide prevalence of approximately 25%, and this is expected to increase in the future due to the increasing number of obese people who have metabolic syndrome.^11^, ^12^

Furthermore, nonalcoholic fatty liver disease is classified into nonalcoholic fatty liver, which has limited pathologic progression, and nonalcoholic steatohepatitis which has a more severe progressive nature.^13^, ^14^ Nonalcoholic fatty liver is a disease with a favorable prognosis, whereas nonalcoholic steatohepatitis can have fatal consequences with the gradual progression of inflammation and fibrosis transitioning into end‐stage liver disease, such as cirrhosis and hepatocellular carcinoma. Therefore, appropriate strategic interventions for the prevention and early treatment of nonalcoholic steatohepatitis are required.^6^, ^7^ However, since the terms nonalcoholic fatty liver disease and nonalcoholic steatohepatitis do not reflect the cause of the disease and encompass numerous clinical conditions, there has been a movement in recent years to further subdivide the disease and develop new nomenclature to change the name of nonalcoholic fatty liver disease and nonalcoholic steatohepatitis to “metabolic fatty liver disease” and “metabolic steatohepatitis.”^6^, ^7^

Recently, there has also been a lively debate over the possible development of a periodontal disease–related nonalcoholic fatty liver disease and nonalcoholic steatohepatitis, which is the main theme of this chapter. Research related to periodontal disease and nonalcoholic fatty liver disease has gradually changed over time. Between the 1990s and the early 2000s, a bidirectional association between poor oral hygiene with the presence of periodontal disease and chronic hepatitis and cirrhosis was suggested.^15^, ^16^, ^17^, ^18^ Later in the 2000s, the possible involvement of systemic inflammation and oxidative stress derived from periodontitis in the development of nonalcoholic fatty liver disease emerged from in vitro–based basic research.^19^, ^20^ Then, in the early 2010s, the possible involvement of *Porphyromonas gingivalis*, a common periodontopathic bacteria, in the development of nonalcoholic fatty liver disease was reported and continues to be discussed to this day.^21^, ^22^, ^23^ Related to this, the concept of a gut‐liver axis and gut dysbiosis was further proposed as another potential route linking the oral cavity and the liver.^24^, ^25^ Since the late 2010s, systematic reviews and meta‐analyses^26^, ^27^, ^28^ have continued to report on these associations based on growing evidence from epidemiologic studies^21^, ^29^, ^30^, ^31^, ^32^, ^33^, ^34^, ^35^, ^36^, ^37^, ^38^, ^39^, ^40^, ^41^, ^42^, ^43^, ^44^, ^45^, ^46^, ^47^, ^48^, ^49^ and on additional evaluation from in vivo research.^50^, ^51^, ^52^, ^53^, ^54^, ^55^, ^56^, ^57^, ^58^, ^59^ Moreover, as a next step, clinical studies with therapeutic intervention are expected to verify the effect of periodontal treatment on nonalcoholic fatty liver disease and nonalcoholic steatohepatitis.^60^, ^61^

The relationship between periodontal disease and nonalcoholic fatty liver disease has been discussed from in vitro, in vivo, and epidemiologic perspectives, although no review has ever discussed these in a systematic manner, which is the aim of the current review. In this review, we provide updates based on current evidence on the pathogenesis, clinical data, and treatment of nonalcoholic fatty liver disease and nonalcoholic steatohepatitis involved with periodontal disease. After providing an explanation of the epidemiology and etiology of nonalcoholic fatty liver disease, the present status of the association between nonalcoholic fatty liver disease and periodontal disease will be presented. We will also explain the interrelationship of metabolic disorders and periodontal disease with nonalcoholic fatty liver disease and will organize the research evidence into the two pathways that link periodontal disease with liver disease, through the hematogenous and enteral routes. Furthermore, specific examples of periodontal disease–derived risk factors that play an important role in nonalcoholic fatty liver disease and nonalcoholic steatohepatitis will be discussed. Lastly, the possibility of periodontal treatment and the future outlook of nonalcoholic fatty liver disease and nonalcoholic steatohepatitis research will be outlined.

As previously mentioned, separately classifying insulin resistance–associated nonalcoholic fatty liver disease and nonalcoholic steatohepatitis^6^, ^7^ from that which is associated with periodontal disease may be a development that emerges in the near future. This would support therapeutic intervention based on a periodontal approach, which may enable early treatment of this life‐threatening liver disease.

## 2 EPIDEMIOLOGY, ETIOLOGY, AND CLINICAL DIAGNOSIS OF NONALCOHOLIC FATTY LIVER DISEASE/NONALCOHOLIC STEATOHEPATITIS

### 2.1 Anatomic features and physiologic role of the liver

The liver is a prominent organ in terms of its metabolism, synthesis, and detoxification functions. It also plays an important role in regulating blood glucose and lipids, and it has the potential to regenerate even after tissue damage.^4^ The central function of the liver in homeostasis and the inflammatory response is made possible by its unique anatomic location; and it is the largest parenchymal organ, receiving a dual blood supply from systemic circulation and the gastrointestinal tract.^5^ The liver receives 80% of its blood supply via the intestinal portal vein, which is rich in bacterial products, environmental toxins, and food antigens. The remaining 20% is derived from the hepatic artery, which is a feeding vessel branching from the abdominal aorta. The blood from the two circulatory systems joins at the hepatic hilum and then spreads throughout the liver via a capillary network called sinusoids. In other words, the liver is the hemodynamic confluence of the human body, and the large amount of blood that continuously flows into the liver through the sinusoids allows for a diverse composition of intrahepatic cell populations comprised of the metabolically active hepatocytes, nonparenchymal hepatocytes, and various immune cells.^62^

In particular, liver function depends on its strong innate immune system to provide effective and rapid protection against potentially toxic substances without causing a harmful immune response.^4^, ^5^ This role includes intrahepatic enrichment of innate immune cells (Kupffer cells, hepatic stellate cells, natural killer, natural killer T, and T cells, etc), immunologic elimination of microorganisms, and removal of waste molecules.^63^ Such complex communication between intrahepatic immune cells and hepatocytes is primarily mediated by cytokines, which activate effector functions of immune cells and hepatocytic intracellular signaling pathways controlling cell homeostasis. Kupffer cells and liver‐infiltrating monocyte‐derived macrophages are major sources of cytokines, such as tumor necrosis factor alpha and interleukin (IL)‐6. Moreover, the biosynthesis of numerous soluble pathogen‐recognition receptors and complement components plays an important role in controlling systemic innate immunity.^5^

However, the liver is susceptible to metabolic and endocrine disorders due to the action of drugs, microorganisms, and environmental factors, and this imbalance can lead to pathologic consequences.^62^ Given its regenerative capacity, the liver can overcome severe damage in many circumstances, but chronic damage progressively promotes a homeostatic imbalance, resulting in various chronic liver diseases, such as steatosis, hepatitis, fibrosis, cirrhosis, and hepatocellular carcinoma.

### 2.2 Disease definition, prevalence, and epidemiology of nonalcoholic fatty liver disease/nonalcoholic steatohepatitis

**FIGURE 1:**
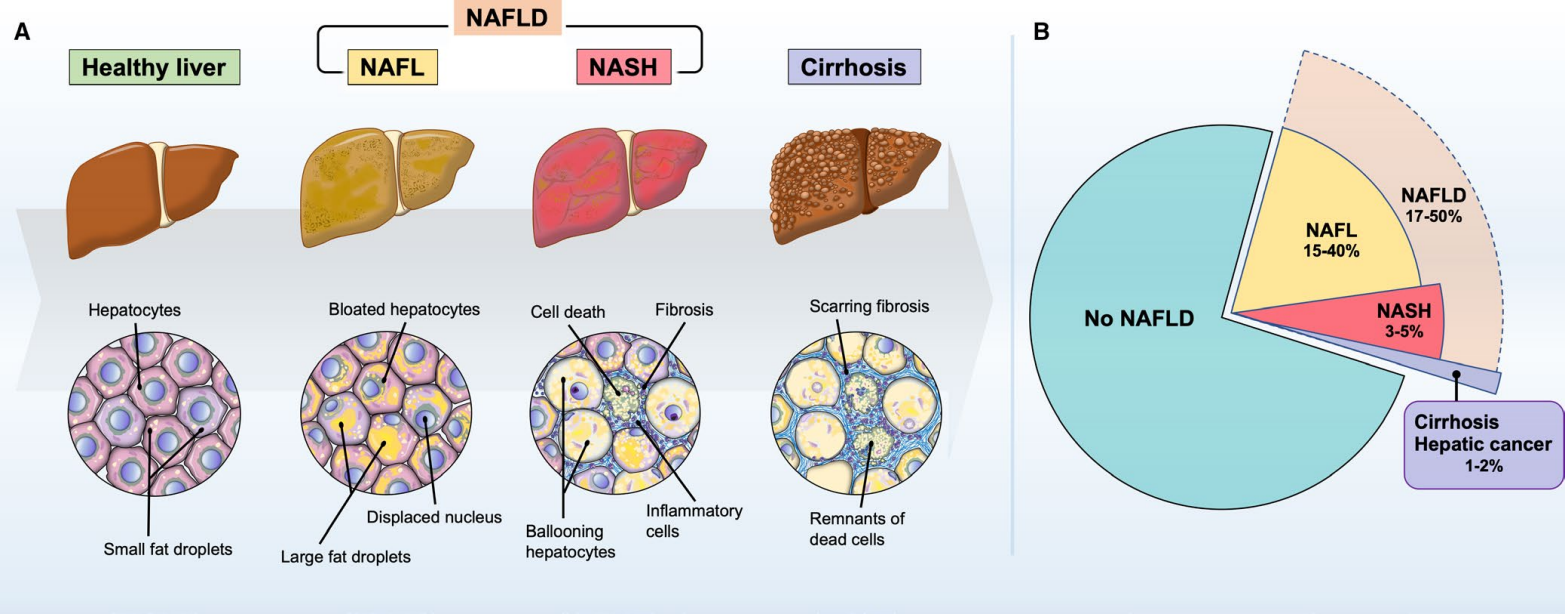
Histologic features and prevalence of nonalcoholic fatty liver disease (NAFLD) and nonalcoholic steatohepatitis (NASH). A, Healthy liver normally contains some fat, but if more than 5% of hepatocytes are fatty, then it is diagnosed as fatty liver or steatosis. The spectrum of nonalcoholic fatty liver disease ranges from nonalcoholic fatty liver (NAFL: simple steatosis) to nonalcoholic steatohepatitis, which can ultimately progress to end‐stage liver disease. In addition to the fatty deposition in the liver, nonalcoholic steatohepatitis is characterized by inflammation, hepatocellular damage, and cell death with or without fibrosis. Furthermore, nonalcoholic steatohepatitis can lead to scarring fibrosis and eventually progress to cirrhosis, hepatic insufficiency, and hepatocellular carcinoma. B, Global prevalence of nonalcoholic fatty liver disease was estimated to be 25% on average in the wide range from 17% to 50% according to the data from Estes et al^11^ and Younossi et al.^12^ Approximately 20% of nonalcoholic fatty liver disease cases would be classified as nonalcoholic steatohepatitis, which represents 3%‐5% of the overall adult population. The worldwide prevalence of nonalcoholic fatty liver disease spectrum and subsequent cirrhosis have been projected to increase greatly by 2030

Nonalcoholic fatty liver disease, which affects both children and adults, is currently the most prevalent chronic liver disease worldwide.^64^ Nonalcoholic fatty liver disease is defined as cases showing the presence of hepatic steatosis (greater than 5% of hepatocytes are fatty) but lacking common causes of secondary hepatic fat accumulation, such as excessive alcohol consumption, chronic viral hepatitis, autoimmune hepatitis, long‐term use of steatosis‐inducing medications, or congenital hepatic disorders.^6^, ^9^, ^65^, ^66^ The majority of nonalcoholic fatty liver diseases are nonalcoholic fatty liver (simple steatosis) with good prognosis (Figure 1), but a subgroup of about 20%‐30% of these patients can develop into more severe and progressives forms of liver disease, namely nonalcoholic steatohepatitis.^9^ Nonalcoholic steatohepatitis is characterized by histologic findings, including, in addition to lipid deposition, inflammatory cell infiltration, ballooning degeneration of hepatocytes, and fibrosis, and it is extremely difficult to distinguish between simple fatty liver and nonalcoholic steatohepatitis using noninvasive examination, such as blood biomarkers and ultrasonography.^67^ Therefore, the gold standard for diagnosing nonalcoholic steatohepatitis remains a liver biopsy and exclusion of secondary causes.^68^ Nonalcoholic steatohepatitis, also known as the liver phenotype of metabolic syndrome, is strongly associated with severe metabolic complications, such as obesity and diabetes mellitus.^8^ Moreover, a portion of nonalcoholic steatohepatitis patients have been reported to progress to cirrhosis and hepatocellular carcinoma, which are end‐stage liver diseases.^13^, ^14^

The prevalence of nonalcoholic fatty liver disease has been estimated to range between 20% and 50%, depending on the study population and diagnostic methods used, and it continues to increase worldwide as the number of obese individuals grows.^69^, ^70^, ^71^ A meta‐analysis study by Younossi et al^12^ revealed that the global prevalence of nonalcoholic fatty liver disease is 25.24%, and is highest in the Middle East and South America, followed by Asia, North America, Europe, and Africa. It has been reported that the annual incidence of nonalcoholic fatty liver disease ranged between 20 and 50 cases per 1000 people in different countries.^6^ Moreover, the overall mortality rate of patients with nonalcoholic fatty liver disease has increased significantly in recent years due to cardiovascular events and liver‐related disorders, wherein the rate of nonalcoholic steatohepatitis patients is higher than that of patients with simple steatosis.^72^, ^73^, ^74^ These surprising facts strongly indicate that nonalcoholic fatty liver disease and nonalcoholic steatohepatitis are at the center of the new pandemic of chronic liver disease, thus mediating a significant clinical and economic burden.^75^, ^76^

### 2.3 Etiology and pathophysiology of nonalcoholic fatty liver disease/nonalcoholic steatohepatitis

The pathogenesis of nonalcoholic fatty liver disease and nonalcoholic steatohepatitis involves multiple factors and processes, such as altered energy metabolism, an altered host immune system, enterobacteria, and genetic predisposition. Until now, the mechanism of its onset and progression has been explained from the perspective of a “two‐hit theory” proposed by Day and James.^77^, ^78^ According to this theory, the first hit involves a sedentary lifestyle, high‐fat diet, obesity, and insulin resistance, which enhance hepatic lipid accumulation and induce a fatty liver, thereby making the liver susceptible to further negative stimuli. Subsequently, it has been presumed that various hepatocyte‐damaging factors, such as proinflammatory cytokines, gut microbiota–derived components, oxidative stress, and lipid peroxide, act as the second hit, leading to necrotic inflammation and fibrosis in the fatty liver. However, a two‐hit theory alone is not sufficient to explain all of the molecular and metabolic alterations occurring in nonalcoholic fatty liver disease, and in some cases it is necessary to assume that inflammation precedes the hepatic steatosis.^79^, ^80^ Therefore, the current widely accepted theory is that of a “multiple parallel hits hypothesis.” This theory explains that there is an interaction between genetic and environmental factors, as well as changes in crosstalk between different organs, including adipose tissue, the intestine, the pancreas, and the liver. Together, this suggests that a more widespread and simultaneous metabolic dysfunction is involved in the process of nonalcoholic fatty liver disease and nonalcoholic steatohepatitis.^81^

### 2.4 Evaluation and diagnosis of nonalcoholic fatty liver disease/nonalcoholic steatohepatitis

The methods for evaluating nonalcoholic fatty liver disease and nonalcoholic steatohepatitis vary from study to study. Representative methods^82^ used in the literature will be discussed in this section.

#### 2.4.1 Pathologic diagnosis

A liver biopsy is the gold standard in the diagnosis of nonalcoholic steatohepatitis (Figure 1). Although the criteria for when to obtain a liver biopsy for nonalcoholic fatty liver disease are not currently established, a hepatic biopsy should be considered if it is difficult to differentiate from other chronic diseases or when nonalcoholic steatohepatitis is suspected. Several pathology‐based classification systems have been employed. Matteoni's criteria^83^ classify nonalcoholic fatty liver disease as type I (steatosis alone), type II (steatosis with lobular inflammation only), type III (steatosis with hepatocellular ballooning), and type IV (type III plus either Mallory‐Denk bodies or fibrosis); plus, types III and IV are diagnostic for nonalcoholic steatohepatitis. Brunt's criteria^84^ evaluate and classify the pathologic findings of nonalcoholic steatohepatitis according to the degree of inflammation (grades 1 to 3) and fibrosis (stages 0 to 4). Also, Kleiner et al^85^ scored liver tissue findings based on the degree of steatosis (score 0 to 3), the degree of lobular inflammation (score 0 to 3), and the frequency of hepatocyte ballooning (score 0 to 2), with total scores of 5 or more for nonalcoholic steatohepatitis, 2 or less for non‐nonalcoholic steatohepatitis as definition of nonalcoholic fatty liver disease, and 3‐4 for borderline cases; the total score is known as the nonalcoholic fatty liver disease activity score. In addition, they defined the stage of fibrosis using a score from 0 to 4, which is evaluated separately from the nonalcoholic fatty liver disease activity score.

#### 2.4.2 Abdominal sonography and computed tomography

Abdominal sonography (ultrasound) has a high detection capability in the presence or absence of moderate or high levels of fat deposits and, therefore, is useful in the diagnosis of nonalcoholic fatty liver disease.^82^, ^86^ However, it is difficult to assess the degree of inflammation and fibrosis.^87^, ^88^ It also cannot be used to differentiate between nonalcoholic fatty liver disease and early nonalcoholic steatohepatitis.^89^, ^90^ With this method, a fatty liver diagnosis was defined as a bright liver, increased liver echotexture compared with the kidneys, vascular blurring, and deep attenuation of the liver.

Abdominal computed tomography is also useful in the diagnosis of nonalcoholic fatty liver disease, and the liver‐to‐spleen ratio can be used to estimate the amount of fat deposition.^82^, ^91^ However, inflammation and fibrosis are difficult to determine by computed tomography, which cannot be used to identify nonalcoholic steatohepatitis.^92^

#### 2.4.3 Blood biomarkers

Serum alanine aminotransferase, aspartate aminotransferase, gamma‐glutamyl transpeptidase, platelets, albumin, triglyceride, cholinesterase, fasting plasma insulin, homeostasis model of assessment of insulin resistance, and other markers have been used as indicators of liver conditions.^82^ Although there are no established biomarkers to detect nonalcoholic steatohepatitis, alanine aminotransferase may be a useful screening method for nonalcoholic fatty liver disease.^93^ However, there is no consensus cutoff value for alanine aminotransferase, and it varies from 40 to 75 IU/L depending on the studies.^94^, ^95^, ^96^ Alanine aminotransferase is also not a good indicator of the severity of the disease. In contrast, the ratio of aspartate aminotransferase to alanine aminotransferase is considered to be an indicator of fibrosis progression, and cutoff values of 1.0 for nonalcoholic steatohepatitis and 0.8 for nonalcoholic fatty liver disease are recommended.

#### 2.4.4 Formula scoring system

Several scoring systems that use a special formula have been developed for the diagnosis and prediction of nonalcoholic fatty liver disease and nonalcoholic steatohepatitis. The nonalcoholic fatty liver disease fibrosis score^97^ is a system used to predict cases of advanced fibrosis. The formula for nonalcoholic fatty liver disease fibrosis score includes age, body mass index, impaired fasting glycemia or diabetes, the aspartate aminotransferase to alanine aminotransferase ratio, platelets, and albumin. The fatty liver index^98^ has been developed to predict the onset of nonalcoholic fatty liver disease, and the formula consists of triglyceride, body mass index, gamma‐glutamyl transpeptidase, and waist circumference. The fatty liver index was further modified for US citizens by taking ethnic differences into consideration.^99^ The hepatic steatosis index^100^ is a system for simplifying the nonalcoholic fatty liver disease evaluation, and the formula consists of alanine aminotransferase to aspartate aminotransferase ratio, body mass index, gender, and diabetes.

## 3 EPIDEMIOLOGIC RELATIONSHIP BETWEEN PERIODONTAL DISEASE AND NONALCOHOLIC FATTY LIVER DISEASE IN HUMANS

The relationship between periodontitis and liver disease has been previously discussed and is based on a growing number of epidemiologic studies. Between the 1990s and the early 2000s, Movin,^101^ Novacek et al,^15^ and Anand et al^17^ investigated the influence of periodontal disease on liver cirrhosis, concluding that poor oral hygiene or poor dental care contributed to the condition rather than it being a direct relationship. Oettinger‐Barak et al^102^ reported greater bone loss in patients with cirrhosis and after liver transplantation than in healthy individuals.

Recently, there has been a focus on the effects of periodontal disease on liver abnormalities, especially on nonalcoholic fatty liver disease. Thus, a literature search was conducted to answer the following question: Does periodontal disease affect the development or progression of nonalcoholic fatty liver disease and nonalcoholic steatohepatitis? To answer that question, the following terms were searched in PubMed/MEDLINE: (periodontitis OR periodontal) AND (hepatic OR liver OR steatosis OR non‐alcoholic fatty liver disease OR nonalcoholic fatty liver disease OR fatty liver OR NAFLD). Furthermore, filters for “Humans,” “English,” and “Adults: 19 years” were used. As a result, we found 154 articles. We excluded studies on viral hepatitis and liver transplantation, case reports, animal studies, and studies with different objectives. We also added six articles using a hand search. Consequently, 13 cross‐sectional studies, two case‐control studies, and three cohort studies were included (Table 1). These will be discussed in the following.

**TABLE 1:** Characteristics of the included studies

Author, year, country	Study design	Number of participants, gender, age	Examiner calibration	Periodontal case definition	Protocol for periodontal examination	Liver disease definition	Analytic approach	Main results	Statistical significance	Conclusion	Reference
Saito et al, 2006, Japan	Cross‐sectional	N = 172 (all participants were females, mean age 40.9 y)	No	Periodontitis subjects having at least one sextant with deepest periodontal probing depth ≥4 mm (code 1 or 2)	Eight designated molars and two incisors with periodontal probing depth recorded	Liver steatosis, hepatic condition Percentage body fat Elevated levels of aspartate aminotransferase, alanine aminotransferase, gamma‐glutamyl transpeptidase, lactate dehydrogenase, alkaline phosphatase, cholinesterase aspartate aminotransferase: ≥32 IU/L alanine aminotransferase: ≥32 IU/L aspartate aminotransferase‐alanine aminotransferase ratio: ≤1 cholinesterase: ≥1.23 (Δ pH)	Age‐adjusted regression analysis Logistic regression models Periodontitis as the dependent variables	Regression coefficient: Aspartate aminotransferase 0.98 Alanine aminotransferase 0.56 Cholinesterase 40 Adjusted odds ratio (95% confidence interval) for periodontitis: Aspartate aminotransferase 4.88 (1.18‐20.21) Alanine aminotransferase 6.79 (1.27‐36.36) Aspartate aminotransferase‐alanine aminotransferase ratio 2.34 (0.99‐5.5) Cholinesterase 3.82 (1.33‐10.96)	Yes	Hepatic steatosis is associated with periodontitis in Japanese women	29
Furuta et al, 2010, Japan	Cross‐sectional	N = 2225 (1264 males and 961 females, aged 18‐19 y)	Yes	Presence of ≥1 teeth with periodontal probing depth ≥4 mm	Randomly selected quadrants, one maxillary and one mandibular with periodontal probing depth and percentage bleeding on probing recorded	Alanine aminotransferase normal: ≤20 IU/L Alanine aminotransferase subclinical: 21‐40 IU/L Alanine aminotransferase abnormal: ≥41 IU/L	Logistic regression analysis Periodontitis as the dependent variables	Adjusted odds ratio (95% confidence interval) for periodontitis: Males, alanine aminotransferase 2.3 (1.0‐5.2) Females, alanine aminotransferase 1.0 (0.1‐9.3)	Yes (for males)	Elevated alanine aminotransferase is a potential risk indicator for periodontitis among healthy young males	30
Yoneda et al, 2012, Japan	Case‐control study	N = 210 (150 nonalcoholic fatty liver disease, 102 nonalcoholic steatohepatitis, and 48 nonalcoholic fatty liver) patients, 64 males and 86 females, mean age of 54.6 y N = 60 healthy subjects, 29 males and 31 females, mean age of 52.9 y	No	Detection of *Porphyromonas gingivalis*, *Treponema denticola*, *Prevotella intermedia*, *Tannerella forsythia*, *Aggregatibacter actinomycetemcomitans*, and *Campylobacter rectus* by polymerase chain reaction technique	Not mentioned	Histopathologic findings (liver biopsy) Steatosis and necroinflammatory activity (criteria of Matteoni et al)	*P. gingivalis–*positive rate (%) Multiple regression analysis: liver disease as the dependent variables Rate of various fimbriae A types on nonalcoholic fatty liver disease patients (%)	*P. gingivalis* (+): non‐nonalcoholic fatty liver disease, 21.7%; nonalcoholic fatty liver disease, 35.4%; nonalcoholic steatohepatitis, 52.0% Adjusted odds ratio (95% confidence interval) for nonalcoholic fatty liver disease: 2.62 (1.00‐6.83) 94.3% of *P. gingivalis*–positive specimens were invasive fimbriae A genotypes	Yes (not for nonalcoholic fatty liver to control)	*P. gingivalis* infection was noted at a significantly high frequency in nonalcoholic fatty liver disease and nonalcoholic steatohepatitis patients	21
	Case‐series	N = 10	No	Patients had a periodontal probing depth of >5 mm in at least four teeth	Not mentioned	Abnormal levels of aspartate aminotransferase and alanine aminotransferase		Level of aspartate aminotransferase and alanine aminotransferase decreased	Yes		
Ahmad et al, 2015, Japan	Cross‐sectional	N = 5477 (4207 males, mean age of 45.4 years and 1270 females, mean age 45.9 y)	Yes, interexaminer	Not mentioned	Mesio‐buccal and mid‐buccal sites for all teeth, except for third molars, with periodontal probing depth and clinical attachment level	Alanine aminotransferase ≥40 IU/L	Multiple regression models Periodontitis as the dependent variables	Mean and standard deviation of periodontal probing depth (mm) in low alcohol consumption group in males Elevated alanine aminotransferase (−), metabolic syndrome (−): 2.09 ± 0.36 Elevated alanine aminotransferase (+), metabolic syndrome (−): 2.12 ± 0.35 Elevated alanine aminotransferase (−), metabolic syndrome (−): 2.18 ± 0.41 Elevated alanine aminotransferase (−), metabolic syndrome (+): 2.21 ± 0.36	Yes	Significant association of liver abnormalities and metabolic syndrome with periodontal condition in males with low alcohol consumption	32
Wiener et al, 2016, USA	Cross‐sectional	N = 5758 (50.1% females, 41.9% 30‐44 y, 28.7% 45‐54 y, 29.4% 55‐69 y)	No	Following American Academy of Periodontology/Centers for Disease Control and Prevention definition: mild periodontitis, moderate periodontitis, severe periodontitis	Not mentioned	Alanine aminotransferase ≥40 IU/L	Logistic regression analysis Periodontitis as a dependent variable	Adjusted odds ratio: 1.17 (0.85‐1.60)	No (yes for unadjusted odds ratio)	Positive but attenuated association of periodontitis and alanine aminotransferase failed to reach significance when other known, strong factors of periodontitis were included in the analysis	34
Akinkugbe et al, 2017, Pomerania	Cohort study	N = 2623 (41% males and 59% females, mean age of 46 y)	No	Proportion of sites with clinical attachment level ≥4 mm or periodontal probing depth ≥3 mm (0%, <30%, ≥30%)	Mesio‐buccal, mid‐buccal, disto‐buccal, and mid‐lingual site for all teeth except for third molars in two quadrants, with periodontal probing depth and clinical attachment level	Abdominal sonography Serum alanine aminotransferase >0.57 μmol/system of units (34.2 IU/L) for men, >0.4 μmol/system of units (24 IU/L) for women Median of 7.7 y incidence	Weighted Poisson regression estimated Median of 7.7 y incidence Incidence rate difference with multiple imputation Liver disease as a dependent variable	Adjusted incidence rate relative to no site of clinical attachment level of 3 mm: <30%: 1.28 (0.84‐1.95) ≥30%: 1.60 (1.05‐2.43) Adjusted incidence rate difference relative to no site of clinical attachment level of 3 mm: <30%: 5.49 (−2.53‐13.5) ≥30%: 11.9 (4.09‐19.6) Adjusted incidence rate relative to no site of periodontal probing depth of 4 mm: <30%: 1.53 (1.00‐2.35) ≥30%: 0.77 (0.44‐1.33) Adjusted incidence rate difference relative to no site of periodontal probing depth of 4 mm: <30%: 14.6 (8.87‐20.4) ≥30%: −6.34 (−13.7‐1.02)	Yes	History of periodontitis as an independent risk factor contributing to nonalcoholic fatty liver disease incidence in a population‐based sample	36
Akinkugbe et al, 2017, Pomerania	Cross‐sectional study	N = 2481 (55% females, mean age of 47 y)	No	Proportion of sites with periodontal probing depth ≥3 mm (0%, <30%, ≥30%)	4 sites per tooth on 2 quadrants	Liver ultrasonography: increase in liver echogenicity	Logistic regression analysis Stratified according to the median value (1.98) for the C‐reactive protein–specific weighted genetic score (wGS_CRP_) and for low (<1 mg), intermediate (1‐3 mg) and high (>3 mg) levels of serum C‐reactive protein Liver disease as a dependent variable	Adjusted prevalence odds ratio (95% confidence interval): Subjects with wGS_CRP_ ≤ 1.98 <30% sites of periodontal probing depth ≥4 mm: 1.08 (0.75‐1.57) ≥30% sites of periodontal probing depth≧4 mm: 1.14 (0.72‐1.80) Subjects with wGS_CRP_ > 1.98 <30% sites of periodontal probing depth ≥4 mm: 1.33 (0.94‐1.89) ≥30% sites of periodontal probing depth≧4 mm: 1.65 (1.07‐2.55) Subjects with serum C‐reactive protein <1 mg <30% sites of periodontal probing depth ≥4 mm: 1.62 (1.00‐2.61) ≥30% sites of periodontal probing depth ≥4 mm: 2.39 (1.32‐4.31) Subjects with serum C‐reactive protein 1‐3 mg: <30% sites of periodontal probing depth ≥4 mm: 1.37 (0.90‐2.08) ≥30% sites of periodontal probing depth ≥4 mm: 0.97 (0.57‐1.66) Subjects with serum C‐reactive protein >3 mg <30% sites of periodontal probing depth ≥4 mm: 0.70 (0.45‐1.10) ≥30% sites of periodontal probing depth ≥4 mm: 1.12 (0.65‐1.93)	Yes (for interaction for serum C‐reactive protein levels)	Periodontitis was positively associated with higher prevalence odds of nonalcoholic fatty liver disease and this relationship was modified by serum C‐reactive protein levels	35
Widita et al, 2017, Japan	Cohort study	N = 265 (133 males and 132 females, mean age of 72.5 y)	Yes, interexaminer	Periodontal probing depth ≥6 mm and clinical attachment level ≥6 mm	Six sites around each tooth	Elevation of aspartate aminotransferase, alanine aminotransferase, and aspartate aminotransferase/alanine aminotransferase ratio in 8 y	Logistic regression analysis Liver disease as a dependent variable Stratified according to smoking status and alcohol drinking habits	Adjusted odds ratio (95% confidence interval) for fatty liver index Aspartate aminotransferase as a dependent variable Periodontal probing depth ≥6 mm: 1.10 (0.99‐1.22) Clinical attachment level ≥6 mm: 1.02 (0.99‐1.05) Alanine aminotransferase as a dependent variable Periodontal probing depth ≥6 mm: 1.10 (1.00‐1.21) Clinical attachment level ≥6 mm: 1.03 (1.00‐1.06) Smokers periodontal probing depth ≥6 mm: 1.20 (1.00‐1.26) Clinical attachment level ≥6 mm: 1.04 (1.00‐1.07)	Yes for alanine aminotransferase levels (significant interaction of alanine aminotransferase with smoking status)	The elevation of alanine aminotransferase levels might be associated with clinical periodontal parameters among with clinical periodontal parameters among non‐institutionalized Japanese elderly, and this association was modified by smoking status	44
Alzawi et al, 2017, USA and UK	Cross‐sectional (population‐based and patient‐based) study	Population‐based study in USA N = 8172 (3796 males and 4376 females, 20‐74 y)	Population‐based study No	Population based 2 sites with periodontal probing depth ≥3 mm from different sextans or serum immunoglobulin G antibodies against 19 bacterial species in 8153 participants aged ≥40 y	Not mentioned	Population‐based study Presence of steatosis on gallbladder ultrasonography Nonalcoholic fatty liver disease fibrosis score	Population‐based study Logistic regression analysis Liver disease as a dependent variable	Population based study Unadjusted odds ratio (95% confidence interval) Bleeding on probing (%): 1.10 (1.04‐1.07) Periodontal probing depth ≥4 mm (%): 1.06 (1.01‐1.10) Mean periodontal probing depth: 1.11 (1.05‐1.18) Clinical attachment level >3 mm (%) : 1.13 (1.06‐1.20) Mean clinical attachment level: 1.12 (1.04‐1.21) Adjusted for demographic socioeconomic and behavioral factors Bleeding on probing (%): 1.07 (1.00‐1.17) Mean periodontal probing depth: 1.11 (1.05‐1.08) Adjusted for demographic socioeconomic factor, behavioral factors, and cholesterol Mean periodontal probing depth: 1.08 (1.00‐1.17) Odds ratio (95% confidence interval) Antibodies of *Selenomonas noxia*: 1.13 Antibodies of *Streptococcus oralis*: 1.14	Yes (for unadjusted model and some adjusted models)	Complementary evidence from an epidemiologic survey and a clinical study show that nonalcoholic fatty liver disease is associated with periodontitis and the association is stronger with significant liver fibrosis	37
		Patient‐based study in UK N = 69 (periodontitis patients: mean age of 49.2 y, no periodontitis patients: mean age of 50.6 y)	Patient‐based study No	Patient‐based study Basic periodontal examination code 3 (periodontal probing depth: 3.5‐5.5 mm) in 2 or more sextant or 4 (periodontal probing depth >5.5 mm) in any sextant	Patient‐based study Not mentioned	Patient‐based study Kleiner criteria (liver biopsy)	Patient‐based study Spearman test Odds ratio, relative risk Periodontitis as a dependent variable	Patient‐based study Liver stiffness (kPa): periodontitis: 15.3, no periodontitis: 8.9 Number of periodontitis patients: 11/38 in nonalcoholic steatohepatitis, 1/31 in nonalcoholic fatty liver Odds ratio (95% confidence interval) Nonalcoholic steatohepatitis to nonalcoholic fatty liver disease: 12.2 (1.48‐101.0) Relative risk (95% confidence interval) Nonalcoholic steatohepatitis and diabetes: 1.54 (1.04‐2.28) Nonalcoholic steatohepatitis without diabetes: 1.14 (0.95‐1.38)	Yes		
Komazaki et al, 2017, Japan	Cross‐sectional	N = 52 with nonalcoholic fatty liver disease, mean age of 55 y	No	Antibody titers against *A. actinomycetemcomitans*, *F. nucleatum*, *P. gingivalis*		Ultrasonography: increase in echoes in the liver Abdominal computed tomography: liver‐spleen ratio, fat area	Spearman test	Correlation coefficient *ρ* Anti–*A. actinomycetemcomitans* immunoglobulin G to total fat area: 0.38 Anti–*F. nucleatum* immunoglobulin G to total fat area: 0.31 Anti–*A. actinomycetemcomitans* immunoglobulin G to visceral fat area: 0.37	Yes	Infection of *A. actinomycetemcomitans* affects nonalcoholic fatty liver disease by altering the gut microbiota and glucose metabolism	38
Nakahara et al, 2018, Japan	Case control study	nonalcoholic fatty liver disease patients N = 200 (106 males and 94 females, mean age of 51.5 y) Non‐nonalcoholic fatty liver disease patients N=? (data has not been provided)	No	Serum immunoglobulin G antibody titers against *P. gingivalis* fimbriae A type 1, 2, and 4		Liver biopsy: criteria of Matteoni, Brunt, and Kleiner Abdominal computed tomography: visceral fat area	Logistic regression analysis Liver disease as a dependent variable	Univariate odds ratio (95% confidence interval) Type 1: 1.81 (0.99‐3.32) Type 2: 1.49 (0.83‐2.67) Type 4: 2.17 (1.12‐3.99)	Yes (for type 4)	*P. gingivalis* infection is an important risk factor for pathologic progression in nonalcoholic fatty liver disease	42
Iwasaki et al, 2018, Japan	Cross‐sectional study	N = 1226 (772 males and 454 females, mean age of 50 y)	Yes, interexaminer	One or more teeth with ≥4 mm periodontal probing depth	Mesio‐bucal, mid‐buccal, disto‐buccal Mesio‐lingual, mid‐lingual, disto‐lingual per tooth (data of subject teeth has not been provided)	Ultrasonography in the absence of other case of chronic liver disease Bright liver, increased liver echotexture with kidneys, a vascular blurring, and deep attenuation of the liver	Logistic regression analysis Liver disease as a dependent variable	Nonalcoholic fatty liver disease prevalence rate (%) significantly increased according to the severity of periodontal disease Odds ratio (95% confidence interval) For all: 1.88 (1.18‐2.99) Males: 1.62 (0.95‐2.78) Females: 2.97 (1.11‐7.98)	Yes (for females)	There appears to be a positive association between ultrasound‐diagnosed nonalcoholic fatty liver disease and having periodontal probing depth ≥4 mm	40
Kuroki et al, 2018, Japan	Cross‐sectional study	N = 110 (66 males and 44 females, mean age of 73.3 y)	Yes, interexaminer	Not mentioned	Mesial and distal sites of alveolar bone loss (percentage of distance between cementoenamel junction to alveolar crest and cementoenamel junction‐apex) for all remaining teeth, including third molars on panoramic radiography	Aspartate aminotransferase >30 IU/L Aspartate aminotransferase >42 IU/L for males Aspartate aminotransferase >23 IU/L for females Gamma‐glutamyl transpeptidase >32 IU/L for females	Logistic regression analysis Liver abnormalities as a dependent variable	Adjusted odds ratio Aspartate aminotransferase: 1.43 (0.46‐4.48) Alanine aminotransferase: 1.24 (0.37‐4.18) Gamma‐glutamyl transpeptidase: 0.95 (0.03‐1.16)	No	There was no significant association between the elevation of serum live enzyme levels and alveolar bone loss in Japanese adults	41
Akinkugbe et al, 2018, USA (Hispanic and Latino)	Cross‐sectional study	N = 11 914 (45.1 males and 54.9% females, mean age of 40.4 y)	No	Percentage of sites (none, <30%, ≥30%) affected by clinical attachment level ≥3 mm or periodontal probing depth ≥4 mm	Not mentioned	Nonalcoholic fatty liver disease Alanine aminotransferase >40 IU/L for males Alanine aminotransferase >31 IU/L or aspartate aminotransferase >37 IU/L for females Fatty liver index score ≥60%	Prevalence odds ratio Liver disease as a dependent variable	Adjusted prevalence odds ratio Clinical attachment level ≥3 mm <30%: 1.03 (0.87‐1.21) ≥30%: 0.91 (0.70‐1.18) Periodontal probing depth ≥4 mm <30%: 1.03 (0.88‐1.20) ≥30%: 1.00 (0.72‐1.38)	No	Previously reported associations between periodontitis and nonalcoholic fatty liver disease were not replicated in a diverse group of Hispanic/Latino men and woman	39
Shin, 2019, South Korea	Cross‐sectional study	N = 4061 (1476 males and 2585 females, >19 y)	No	Presence of periodontal pockets (community periodontal index score 3‐4)	10 index teeth: the first and second molars, the upper right incisor, and the lower left incisor	Fatty liver index score >60% Hepatic steatosis index >36	Chi‐square test Generalized linear model Liver disease as a dependent variable	Prevalence (%) of nonalcoholic fatty liver disease for women 1) In fatty liver index ≥60 subjects 1‐1) No periodontal pockets: 4.6 1‐2) Periodontal pockets: 13.2 2) In hepatic steatosis index ≥36 subjects 2‐1) No periodontal pockets: 15.9 2‐2) Periodontal pockets: 29.5 3) Adjusted odds ratio (95% confidence interval) for fatty liver index for women 3‐1) Mild periodontitis: 1.51 (0.78‐2.91) 3‐2) Severe periodontitis: 2.05 (1.20‐3.52) 4) Adjusted odds ratio (95% confidence interval) for HIS for women 4‐1) Mild periodontitis: 1.89 (1.13‐3.16) 4‐2) Severe periodontitis: 1.40 (0.88‐2.24)	Yes	Significant association between the presence of periodontal pockets measured by community periodontal index and nonalcoholic fatty liver disease in the Korean population	49
Weintraub et al, 2019, USA	Cross‐sectional	N = 5421 (47.9% males and 52.1% females, 21‐71 y)	No	Moderate periodontitis ≥2 teeth with clinical attachment level ≥4 mm or periodontal probing depth ≥5 mm at interproximal Severe periodontitis ≥2 teeth with clinical attachment level ≥6 mm and ≥1 tooth with periodontal probing depth ≥5 mm at interproximal	Not mentioned	Nonalcoholic fatty liver disease Ultrasonography: moderate to severe hepatic steatosis Nonalcoholic fatty liver disease fibrosis score ≥−1.455 Fatty liver index ≥30 US fatty liver index ≥30	Logistic regression analysis Liver disease as a dependent variable	Odds ratio (95% confidence interval) Nonalcoholic fatty liver disease assessed by Ultrasonography :1.54 (1.06‐2.24) Nonalcoholic fatty liver disease fibrosis score : 3.10 (2.31‐4.17) Fatty liver index: 1.61 (1.13‐2.28) US fatty liver index: 2.21 (1.74‐2.98)	Yes	Nonalcoholic fatty liver disease was significantly associated with tooth loss, moderate to severe periodontitis, and for some nonalcoholic fatty liver disease measures, untreated caries, after adjusting for several key sociodemographic factors	46
Helenius‐Hietala et al, 2019, Finland	Cohort study	N = 6165 (45.3% males and 54.7% females, mean age of 49.5 y)	Yes	At least one tooth with a periodontal pocket at least 4 mm deep; Mild to moderate periodontitis: 1‐4 teeth with ≥4 mm deep periodontal pockets Advanced periodontitis: ≥5 teeth with ≥4 mm deep periodontal pockets	Each tooth excluding wisdom teeth on four surfaces	Nonalcoholic fatty liver disease (for baseline): Fatty liver index >60 with alcohol use <30 g/d for men or <20 g/d for women 13‐y incidence as follows: First hospitalization owing to liver disease Liver‐related death Diagnosis of primary liver cancer	Cox model; hazard ratio Severe liver event as a dependent variable	Adjusted hazard ratio (95% confidence interval) Mild periodontitis: 2.24 (0.98‐4.84) Advanced periodontitis: 3.29 (1.53‐7.05) Adjusted hazard ratio (95% confidence interval) in baseline nonalcoholic fatty liver disease patient Mild periodontitis: 3.23 (0.62‐16.8) Advanced periodontitis: 6.94 (1.43‐33.6)	Yes (for advanced periodontitis)	Epidemiologic link independent of multiple confounders beween periodontitis and incident severe liver disease were found	45
Kim et al, 2020, South Korea	Cross‐sectional study	N = 4272; 1113 with periodontitis (51.7% males and 48.3% females), mean age of 53.1 y, and 3159 of nonperiodontitis (38.9% males and 61.1% females), mean age of 41.2 y	No	Community periodontal index score 3 and 4	10 index teeth: the first and second molars, the upper right incisor, and the lower left incisor	Fatty liver index divided by quartile	Logistic regression analysis	Adjusted odds ratio (95% confidence interval) 2nd quartile of fatty liver index: 1.29 (0.97‐1.71) 3rd quartile of fatty liver index: 1.43 (1.06‐1.93) 4th quartile of fatty liver index: 1.63 (1.24‐2.16)	Yes	Fatty liver index may be associated with periodontitis prevalence, especially in subjects with diabetes	48

### 3.1 Cross‐sectional studies

#### 3.1.1 Studies using biomarkers as an indicator of liver abnormalities

Early studies of the association between periodontal disease and liver abnormalities using blood biomarkers were conducted mainly in Japan. Saito et al^29^ studied the association between periodontitis and liver status in 172 women with an average age of 40.9 years who attended a health promotion program. The results showed that age‐adjusted regression coefficients of serum aspartate aminotransferase, alanine aminotransferase, lactate dehydrogenase, gamma‐glutamyl transpeptidase, cholinesterase, high‐density lipoprotein cholesterol, fasting blood glucose, blood cell count, total protein, and urea were significantly associated with the severity of periodontitis. The levels of aspartate aminotransferase, alanine aminotransferase, gamma‐glutamyl transpeptidase, lactate dehydrogenase, alkaline phosphatase, and cholinesterase in serum were significantly higher in patients with periodontitis than in nonperiodontitis patients. A linear multiple regression analysis was performed using data from these blood tests as independent variables, adjusted for age, smoking history, and oral hygiene; the results showed that serum aspartate aminotransferase, alanine aminotransferase, gamma‐glutamyl transpeptidase, cholinesterase, lactate dehydrogenase, and high‐density lipoprotein cholesterol (inversely proportional) were significantly correlated with the severity of periodontitis. Logistic regression analysis showed significant odds ratios for serum alanine aminotransferase, aspartate aminotransferase to alanine aminotransferase ratio, and cholinesterase for periodontitis (probing pocket depth of 4 mm and over) incidence with or without adjustment for body mass index, age, smoking history, oral hygiene, and/or body fat percentage.

In another region of Japan, Furuta et al^30^ conducted a cross‐sectional study of the relationship between periodontal disease and liver abnormalities in 2225 students that were 18 to 19 years of age. In male subjects, normal serum alanine aminotransferase levels (less than 20 IU/L) were observed in 95.8% of nonperiodontitis patients and in 4.2% of periodontitis patients, whereas abnormal levels were found in 87.4% of nonperiodontitis patients and in 12.6% of periodontitis patients. These differences between normal and abnormal levels were statistically significant. When using logistic regression analysis, males were significantly more likely to have periodontitis if their serum alanine aminotransferase was high (greater than or equal to 41 IU/L) than if it was low (adjusted odds ratio of 2.3). However, no significant relationship was found in females. These results differ from those of Saito et al, who found an association between periodontitis and liver abnormalities in females.

In addition, Ahmad et al^32^ investigated the association between hepatic abnormality, metabolic syndrome, and periodontal status in 5477 employees of a manufacturing company in Japan. They found that the mean probing pocket depth in the low alcohol consumption group with higher alanine aminotransferase and metabolic syndrome was significantly higher than the mean probing pocket depth in the normal alanine aminotransferase without metabolic syndrome group. However, no difference was found in females, which is partly consistent with the results of Furuta et al. Differences in the age of the participants, cutoff values for the biomarkers, and/or periodontal examination protocols might explain the differences in the results from the study by Saito et al, which found an association in the female subjects.

A similar study was subsequently conducted in the United States. Wiener et al^34^ investigated the association between periodontitis and alanine aminotransferase in 5758 individuals, 30‐69 years of age, from the 2009‐2010 and 2011‐2012 National Health and Nutrition Examination Survey databases. The criteria for periodontitis that were used as a dependent variable were mild periodontitis, moderate periodontitis, and severe periodontitis based on the definition of the American Academy of Periodontology and Centers for Disease Control and Prevention. Serum alanine aminotransferase was set at 40 IU/L as a cutoff value. Sociodemographic and behavioral variables were also analyzed as cofounding factors. The percentage of periodontitis patients with serum alanine aminotransferase greater than or equal to 40 IU/L and less than 40 IU/L were 38.2% and 39.2%, respectively. Logistic regression analysis showed that the adjusted odds ratio was 1.17 for alanine aminotransferase greater than or equal to 40 IU/L, which was not statistically significant when periodontitis was the dependent variable. The variation in ethnicity (American vs Japanese population) may have contributed to the different results.

Kuroki et al^41^ recently studied the relationship between the levels of serum biomarkers (aspartate aminotransferase, alanine aminotransferase, and gamma‐glutamyl transpeptidase) and alveolar bone (assessed from panoramic radiographs) in 110 residents (mean age 73.3 years) on a Japanese island. Participants were divided into quartiles according to individual values of alveolar bone loss. The frequency of subjects who have the highest alveolar bone loss quartile was not significantly different between those with below and above normal levels of aspartate aminotransferase, alanine aminotransferase, and gamma‐glutamyl transpeptidase. Further, the results from multiple logistic regression analysis with blood parameters as the dependent variable and highest bone loss quartiles as the independent variable showed no significant correlations (adjusted odds ratios of 1.43 for aspartate aminotransferase, 1.24 for alanine aminotransferase, and 0.94 for gamma‐glutamyl transpeptidase). The data obtained in this study were limited to measurements on radiographs and biomarkers in blood samples, which may have prevented the authors from finding a relationship.

#### 3.1.2 Studies using imaging and/or scoring systems to diagnosis nonalcoholic fatty liver disease

The studies described so far have primarily used serum biomarkers as indicators of abnormalities in the liver, and in most cases, multivariate analyses have been performed with periodontal parameters as the dependent variable. However, the direction of research in cross‐sectional studies has now focused on using periodontal disease parameters as the independent variable and liver disease parameters as the dependent variable. Accordingly, in addition to serum biomarkers, other diagnostic methods have been used as parameters of liver disease.

Alazawi et al^37^ investigated the association between periodontitis and nonalcoholic fatty liver disease in two groups: a population‐based study in the United States and a patient‐based study in the UK. Data from the United States National Health and Nutrition Examination Survey III were used for the population‐based study. Periodontitis was defined as the presence of two or more sites with probing pocket depth of 3 mm or sites of 5 mm or more. Nonalcoholic fatty liver disease was defined using the nonalcoholic fatty liver disease fibrosis score. Although nonalcoholic fatty liver disease was significantly correlated with several periodontal parameters, only the mean probing pocket depth remained significant after adjustment for confounding factors. Furthermore, the percentage of subjects with a clinical attachment level of 3 mm or more were 7.5% in the low nonalcoholic fatty liver disease fibrosis score group and 14.7% in the moderate or higher nonalcoholic fatty liver disease fibrosis score group, and this difference was statistically significant. Similarly, the mean clinical attachment level was significantly higher in the group with moderate or higher nonalcoholic fatty liver disease fibrosis score. The patient‐based study in the UK included 69 patients with a mean age of 49.2 years. Periodontitis was defined as the presence of a site with probing pocket depth 3.5‐5.5 mm in more than two sextants or probing pocket depth greater than 5.5 mm. Nonalcoholic fatty liver disease was diagnosed according to Kleiner's criteria from the National Institutes of Health nonalcoholic steatohepatitis clinical research network. In patients with nonalcoholic steatohepatitis with fibrosis score of 2‐4, the percentage of periodontitis patients was 33%, compared with 3% in nonalcoholic fatty liver (simple steatosis) patients. The presence of periodontitis in nonalcoholic steatohepatitis patients (11 out of 38) was significantly higher than that in nonalcoholic fatty liver patients (1 out of 31).

Akinkugbe et al^39^ studied 11914 Hispanics and Latinos (mean age 40.4 years) living in the United States. The results showed no significant correlation between a percentage of clinical attachment level of 3 mm or more or a probing pocket depth of 4 mm or more and serum alanine aminotransferase or aspartate aminotransferase levels and fatty liver index in any of the Mexican, Cuban, Puerto Rican, Dominican, Central American, or South American ethnic groups. The odds ratio for greater than 30% sites with probing pocket depth of 4 mm or more and a clinical attachment level of 3 mm or more was 0.25‐2.22 without adjustment and 0.19‐1.77 with adjustment. These results suggest that a relationship between periodontitis and nonalcoholic fatty liver disease may not be found in some ethnic groups.

On the other hand, Weintraub et al^46^ conducted a population‐based study using data from the National Health and Nutrition Examination Survey III in the United States; 5421 individuals aged 21 to 71 years were included in the study. Logistic regression analysis was used to analyze the relationship between moderate and severe periodontitis, untreated caries, experience of caries, and tooth loss in relation to nonalcoholic fatty liver disease after adjusting for socioeconomic factors. Nonalcoholic fatty liver disease was assessed using four criteria: ultrasonography, nonalcoholic fatty liver disease fibrosis score, fatty liver index, and US fatty liver index. The odds ratios for periodontitis were 1.54 using ultrasonography, 3.10 for nonalcoholic fatty liver disease fibrosis score, 1.61 for fatty liver index, and 2.21 for US fatty liver index. The US fatty liver index is the only scoring system that takes into account ethnic differences, and using this system might help reveal a relationship that was not appreciable in the study by Akinkugbe et al.^39^

In another study in Japan, Iwasaki et al^40^ recruited 1226 subjects with a mean age of 50 years who attended a university hospital for check‐up examinations. The frequency of periodontitis as defined by a probing pocket depth of 4 mm or more was 86.7% in nonalcoholic fatty liver disease patients diagnosed with ultrasonography and 72.9% in non‐nonalcoholic fatty liver disease patients. The frequency of nonalcoholic fatty liver disease was significantly higher in patients with a probing pocket depth of 4‐5 mm or of 6 mm or more compared with patients with a probing pocket depth of less than 3 mm. The odds ratio for all patients with a probing pocket depth of of 4 mm or more was 1.88, which was statistically significant. Also, the odds ratio was 1.62 for males and 2.97 for females, with a significant difference only in females.

Recently, two studies on South Korean populations have been reported. In a population‐based study, Shin^49^ studied 4061 subjects over 19 years of age. Participants with a community periodontal index score of 3 or 4 were defined as having periodontitis, and a diagnosis of nonalcoholic fatty liver disease was made if the fatty liver index was greater than 60 or the hepatic steatosis index was greater than 36. Correlations between periodontitis and nonalcoholic fatty liver disease were then analyzed. The results showed that males with periodontal pockets had significantly higher unadjusted means of fatty liver index than those without periodontal pockets did, but there were no significant differences in adjusted means for the fatty liver index, frequency of fatty liver index less than 60, or means for hepatic steatosis index. In females, however, statistically significant differences were found for all parameters. The odds ratio was not significantly different for males. But for females, the odds ratio for severe periodontitis was 4.27 based on the fatty liver index in the unadjusted case and the adjusted odds ratio ranged from 2.31 to 20.5, with a significant correlation. The odds ratio was 1.40 with the most stringent adjustment based on the hepatic steatosis index, which was no longer significantly different. Another cross‐sectional study in South Korea was carried out by Kim et al.^48^ Using data from the 2010 Korea National Health and Nutrition Examination Survey, a total of 4272 patients were included in the study, of which 1113 had periodontitis. There was a significant difference in the average fatty liver index for periodontitis patients versus nonperiodontitis patients, with means of 21.6 and 12.2, respectively. The percentages of first, second, third, and fourth quartiles for the fatty liver index in patients with periodontitis were 15.9%, 22.2%, 27.8%, and 34.1%, respectively, and the same values in nonperiodontitis patients were 31.0%, 25.9%, 21.8%, and 21.4%, respectively. The adjusted odds ratio for the fatty liver index in all patients was 1.29 in the second quartile, 1.43 in the third quartile, and 1.63 in the fourth quartile. Among them, the fourth quartile adjusted odds ratio was 1.44 in nondiabetic patients and 2.89 in diabetic patients, all of which were statistically significant. The frequency of community periodontal index scores 3 and 4 was highest in the 4th quartile of the fatty liver index. Although the parameters and analysis methods used differed, the results from two Korean population‐based cross‐sectional studies have shown some relationships between periodontitis and nonalcoholic fatty liver disease.

#### 3.1.3 Studies investigating the relationship between nonalcoholic fatty liver disease and other markers associated with periodontal disease

Several cross‐sectional studies have investigated the association between nonalcoholic fatty liver disease and putative periodontopathic bacteria. Komazaki et al^38^ studied 52 patients with nonalcoholic fatty liver disease in Japan and analyzed the correlation between three periodontal bacteria and clinical or biochemical parameters. The results showed that anti–*Aggregatibacter actinomycetemcomitans* antibodies had a significant positive correlation with total fat area, visceral fat area, fasting plasma insulin, a homeostasis model of assessment of insulin resistance, and aspartate aminotransferase, but not with alanine aminotransferase or gamma‐glutamyl transpeptidase. There was a significant negative correlation with the liver‐spleen ratio when assessed by abdominal computed tomography. Anti–*Fusobacterium nucleatum* antibodies had a significant correlation only with total fat area. However, anti–*P. gingivalis* antibodies did not correlate with any liver parameters.

Akinkugbe et al^35^ studied whether serum C‐reactive protein and weighted genetic C‐reactive protein scores (representing cumulative effects of multiple gene loci), which represent the inflammation‐induced burden, affect the relationship between periodontitis and nonalcoholic fatty liver disease. A total of 2481 participants in the West Pomerania region of northeast Germany (the Study of Health in Pomerania) were included in the study. Periodontitis was classified as 0%, less than 30%, and 30% or more of sites with a probing pocket depth of 4 mm or more, and nonalcoholic fatty liver disease was assessed by ultrasonography. Serum C‐reactive protein levels were assessed from blood samples, and a calculation of weighted genetic C‐reactive protein scores was performed. The prevalence of nonalcoholic fatty liver disease was 26.4% overall, 18.1% in subjects with 0% of sites with probing pocket depth of 4 mm or more, 26.6% in the less than 30% group, and 39.2% in the 30% or more group. Periodontitis and nonalcoholic fatty liver disease were correlated with the level of serum C‐reactive protein, but there was no significant association with weighted genetic C‐reactive protein scores. Furthermore, when C‐reactive protein was less than 1 mg/L, the adjusted prevalence odds ratio for nonalcoholic fatty liver disease for the 30% or more sites was 2.39, while the ratio for C‐reactive protein 1‐3 mg/L and greater than 3 mg/L was 0.97 and 1.12, respectively. In other words, there was a significant association between periodontitis and nonalcoholic fatty liver disease in subjects with low levels of C‐reactive protein, but no relationship was found at higher levels of C‐reactive protein. Based on these findings, the authors concluded that serum C‐reactive protein may be a modifier of the relationship between periodontitis and nonalcoholic fatty liver disease. This finding may explain some of the variations in the relationship between periodontitis and nonalcoholic fatty liver disease.

### 3.2 Case‐control studies

Yoneda et al^21^ investigated the association between nonalcoholic fatty liver disease and infection by *P. gingivalis*, which is considered a putative periodontal pathogen. A total of 150 nonalcoholic fatty liver disease patients with mean age 54.6 years and 60 socioeconomically matched healthy individuals (non‐nonalcoholic fatty liver disease; mean age 52.9 years) were included in the study. Nonalcoholic fatty liver disease patients were biopsied and classified according to the criteria of Matteoni et al.^83^ Saliva samples were collected and then various periodontopathic bacteria, including *P. gingivalis*, were quantified by polymerase chain reaction. The detection rate of *P*. *gingivalis* was 52.0% for nonalcoholic steatohepatitis, 35.4% for nonalcoholic fatty liver disease, and 21.7% for controls (non‐nonalcoholic fatty liver disease), with a significant difference between nonalcoholic steatohepatitis and controls. Multiple regression analysis with nonalcoholic fatty liver disease as the dependent variable showed a statistically significant odds ratio of 2.62 for detecting *P. gingivalis*. Most of the *P. gingivalis* fimbriae detected in the nonalcoholic fatty liver disease patients were of invasive genotypes, especially type II (50.0%). The study also included a single‐arm intervention without a control group, and periodontal treatment improved aspartate aminotransferase and alanine aminotransferase.

Nakahara et al^42^ analyzed data from 200 patients with an average age of 51.5 years who were diagnosed with nonalcoholic fatty liver disease by biopsy. Healthy subjects with normal aspartate aminotransferase and alanine aminotransferase data were used as a control group. Serum immunoglobulin G antibody titers against *P. gingivalis* fimbriae A types 1, 2, and 4 were measured. Types 1 and 4 antibody titers tended to be significantly higher in the cases with advanced fibrosis. In particular, the type 4 antibody titers were higher in the advanced stages of nonalcoholic steatohepatitis. The univariate odds ratios for types 1, 2, and 4 were 1.81, 1.49, and 2.17, respectively, and the multivariate odds ratios for types 1 and 4 were 1.08 and 2.08, respectively. Only type 4 showed statistically significant differences. Taken in aggregate, the clinical and translational studies suggest that there is an association between periodontal pathogens and nonalcoholic fatty liver disease.

### 3.3 Cohort studies

A population‐based cohort study was performed using the Study of Health in Pomerania: data from Germany. Akinkugbe et al^36^ included 2623 non‐nonalcoholic fatty liver disease subjects aged 20‐74 years. Subjects were divided into 0%, less than 30%, and 30% or more of sites with 3 mm or more clinical attachment level or 4 mm or more probing pocket depth at baseline. The liver conditions after more than 5 years (median 7.7 years) were investigated by sonography and serum alanine aminotransferase. Relative to subjects without a clinical attachment level of 3 mm or more, the nonalcoholic fatty liver disease incidence was elevated in participants with both less than 30% and 30% or more of sites affected. The adjusted incidence rate ratio for nonalcoholic fatty liver disease was statistically significant at 1.28 for less than 30% of sites and 1.60 for 30% or more of sites affected, respectively. Similarly, the incidence difference was 5.49 for less than 30% of sites and 11.11 for 30% or more of sites affected with a statistically significant difference. On the other hand, no such dose‐response relationship was observed for the probing pocket depth of 4 mm or more. In addition, in patients showing a clinical attachment level of 3 mm or more, the unadjusted incidence rate ratio for 1 mm or more of attachment loss during the observation period was 1.78, and that for 2 mm or more was 2.32, with statistical significance, but it did not reach the level of significance when adjusted. Thus, the authors of this study suggested that a history of periodontitis may be a risk factor for nonalcoholic fatty liver disease.

Helenius‐Hietala et al^45^ conducted a population‐based cohort study in Finland that surveyed 6165 individuals (mean age 49.5 years) in the Finnish population‐based Health 2000 survey. Patients were categorized at baseline as having nonperiodontitis, mild to moderate periodontitis, or severe periodontitis. Participants were also examined for a history of nonalcoholic fatty liver disease, including incident severe liver disease (first hospitalization for liver disease, death from liver disease, and liver cancer) over a 13‐year period. The analysis showed a positive correlation between the number of pockets of 4 mm or more and the hazard ratio of incident severe liver disease. The adjusted hazard ratio for mild to moderate periodontitis was 2.17, which was not statistically significant. On the other hand, the adjusted hazard ratio for severe periodontitis was 3.29, which was statistically significant. In participants who did not have nonalcoholic fatty liver disease at baseline, the hazard ratio for severe periodontitis was 2.09, which was not statistically significant, but the hazard ratio for severe periodontitis was 6.94 in those who had nonalcoholic fatty liver disease, which was statistically significant.

Widita et al^44^ reported on a cohort study of 265 noninstitutionalized Japanese elderly people over 72 years of age. From baseline to 8 years, oral examinations, including a periodontal examination, were performed annually. In addition, blood aspartate aminotransferase and alanine aminotransferase were measured. The number of sites with a probing pocket depth of 6 mm or more or a clinical attachment level of 6 mm or more at baseline was the independent variable, and the increase or decrease in aspartate aminotransferase or alanine aminotransferase over 8 years was the dependent variable, and these relationships were analyzed using logistic regression analysis, which was adjusted for confounding factors. The relationships were also analyzed for individuals that smoked and consumed alcohol. Analysis showed that increased alanine aminotransferase was significantly correlated with periodontal parameters, with an adjusted odds ratio of 1.10 for a probing pocket depth of 6 mm or more and 1.03 for a clinical attachment level of 6 mm or more. However, there was no correlation with aspartate aminotransferase. In subjects with smoking habits but not drinking habits, alanine aminotransferase correlated significantly with probing pocket depth of 6 mm or more (adjusted odds ratio 1.20) and clinical attachment level of 6 mm or more (adjusted odds ratio 1.04).

### 3.4 Systematic review and meta‐analyses

One systematic review and two meta‐analyses have been published on the relationship between periodontitis and nonalcoholic fatty liver disease (Table 2). Alakhali et al^26^ discussed 12 articles (N = 53384), and all but one of them found a significant correlation between periodontal or bacteriologic parameters and nonalcoholic fatty liver disease. The quality of the papers included was also assessed based on the Strengthening of Reporting of Observational Studies in Epidemiology guidelines, with four papers scoring 7, the highest points possible, four scoring 6, and the others 4‐5, which can be considered good. However, the authors did not perform any statistical analysis, such as a meta‐analysis, due to heterogeneity and inconsistency among the studies included.

**TABLE 2:** Summary of the systematic review and meta‐analyses

Author, year	Research question or objective	Database searched	Search period	Language	Study design of included studies	Meta‐analysis	Heterogeneity	Risk of bias assessment tools	Publication bias	Main results	Main conclusion	Reference
Alakhali et al, 2018	Is periodontal disease a potential risk factor for nonalcoholic fatty liver disease?	PubMed/MEDLINE, Scopus, Embase and Web of Science	Up to May 30, 2018	English	9 cross‐sectional studies, 1 cohort study, 1 case‐control study, and 1 case report	No	High (value was not reported)	Strengthening of Reporting of Observational studies in Epidemiology–based quality analysis	Not mentioned	All studies except one found significant associations between clinical and/or microbial periodontal parameters and nonalcoholic fatty liver disease	Periodontitis may be a risk factor for development and progression of nonalcoholic fatty liver disease	26
Wijarnpreecha et al, 2020	To compare the risk of nonalcoholic fatty liver disease among patients with periodontitis versus individuals without periodontitis, by identifying all relevant studies and combining their results together	Ovid MEDLINE and EMBASE	Up to December 2019	No limitation	1 cohort study and 4 cross‐sectional studies	Yes	Based on periodontal probing depth: high for unadjusted odds ratio (*I* ^2^ = 94%, *P* < 0.00001) moderate for adjusted odds ratio (*I* ^2^ = 67%, *P* = 0.02) Based on clinical attachment level: not significant for unadjusted odds ratio (*I* ^2^ = 0%, *P* = 0.88) moderate for adjusted odds ratio (*I* ^2^ = 58%, *P* = 0.09)	Newcastle‐Ottawa quality assessment scale for cohort studies and case‐control studies Modified version of Newcastle‐Ottawa quality assessment scale for cross‐sectional studies	No evidence	When periodontal probing depth >3.5‐4 mm was used as independent variable, pooled unadjusted odds ratio of 1.48 (95% confidence interval: 1.15‐1.89) decreased to 1.13 (95% confidence interval: 0.95‐1.35) and lost its significance. When clinical attachment level >3 mm was used as independent variable, pooled unadjusted odds ratio of 1.13 (95% confidence interval: 1.07‐1.20) deceased to 1.08 (95% confidence interval: 0.94‐1.24) and lost significance	Metabolic conditions, not periodontitis itself, were the predisposing factor for nonalcoholic fatty liver disease	28
Chen et al, 2020	To evaluate whether periodontal disease and tooth loss are associated with liver disease, including nonalcoholic fatty liver disease, liver cirrhosis, liver cancer and other chronic liver disease	PubMed and Embase	Up to March 2020	Not mentioned	Association between periodontitis and nonalcoholic fatty liver disease: 3 cross‐sectional studies and 2 cohort studies Association between periodontitis and elevated transaminase level: 2 cohort studies	Yes	Association between periodontitis and nonalcoholic fatty liver disease: Not significant (*I* ^2^ = 48.5%, *P* = 0.10) Association between periodontitis and elevated transaminase level: not significant (*I* ^2^ = 0%, *P* = 0.37)	Not mentioned	No evidence	Positive associations between periodontal disease and nonalcoholic fatty liver disease (odds ratio 1.19, 95% confidence interval: 1.06‐1.33), and elevated transaminase level (odds ratio 1.08, 95% confidence interval: 1.02‐1.15)	There are positive associations between periodontal disease and nonalcoholic fatty liver disease risk	27

In a review by Wijarnpreech et al,^28^ five papers that met their inclusion criteria were selected. The unadjusted odds ratio for periodontitis with a probing pocket depth of 3.5‐4 mm or more was statistically significant at 1.48 (95% confidence interval 1.15‐1.89), but the adjusted odds ratio decreased to 1.13 (95% confidence interval 0.95‐1.35) and the statistical significance was lost. The unadjusted odds ratio for periodontitis with clinical attachment level of 3 mm or more was significant at 1.13 (95% confidence interval 1.07‐1.20), whereas the adjusted odds ratio was 1.08 (95% confidence interval 0.94‐1.24) and the statistical significance was lost.

Chen et al^27^ published a meta‐analysis of the association between periodontitis and tooth loss and liver disease. Five papers were selected to evaluate the relationship between periodontitis and nonalcoholic fatty liver disease. Since the heterogeneity between the studies was not significant, a meta‐analysis was performed; a significant correlation was found, with an odds ratio of 1.19 (95% confidence interval 1.06‐1.33). The odds ratio decreased to 1.16 (95% confidence interval 1.03‐1.30) when one highly heterogeneous study was excluded. Nonalcoholic fatty liver disease diagnosed by ultrasonography and assessed by the US fatty liver index also showed a significant correlation. They further noted that a similar tendency was maintained even when adjusting for sample size, smoking, alcohol consumption, body mass index, or diabetes.

### 3.5 Summary of epidemiologic studies

Most evidence on the association between periodontitis and nonalcoholic fatty liver disease has been from cross‐sectional studies. Although significant associations have been found in most studies, results have varied, likely due to differences in age, gender,^29^, ^30^ and ethnicity.^39^ In some cases, the significance of the association may have disappeared after adjusting for confounding factors, and a more detailed analysis of the factors and their synergistic effects on the association is necessary. Although cross‐sectional studies alone do not reveal a causal relationship, three cohort studies^36^, ^44^, ^45^ suggested that periodontitis is a potential risk factor for nonalcoholic fatty liver disease.

In addition, it has been suggested that *P. gingivalis* is involved in the progression of nonalcoholic fatty liver disease and nonalcoholic steatohepatitis.^21^, ^42^ Furthermore, a study by Komazaki et al^38^ in conjunction with findings in mice suggests that *A. actinomycetemcomitans* may contribute to nonalcoholic fatty liver disease by altering the bacterial flora and glucose metabolism. However, none of these studies documented clinical parameters, such as probing pocket depth or clinical attachment level, so further studies are needed to determine the mechanisms by which bacteria are involved in nonalcoholic fatty liver disease and nonalcoholic steatohepatitis.

Early publications^29^, ^30^, ^32^ primarily used blood samples to assess nonalcoholic fatty liver disease, but their accuracy was limited, especially in assessing the severity of disease. Using a biopsy is the most useful method for the diagnosis of nonalcoholic steatohepatitis, but it is not practical for repeated assessments because it is an invasive test. Thus, imaging and scoring systems offer advantages to these other methods.

The relationship between periodontal disease and nonalcoholic fatty liver disease has been obtained primarily from observational studies. Although one study^21^ suggested that nonsurgical periodontal treatment reduced *P. gingivalis* levels and improved liver health, the effect of periodontal treatment on liver disease is still largely unknown. Future randomized controlled trials on this topic will be needed to validate this claim.

## 4 RELATIONSHIP BETWEEN PERIODONTAL DISEASE, NONALCOHOLIC FATTY LIVER DISEASE/NONALCOHOLIC STEATOHEPATITIS, AND METABOLIC SYNDROME

**FIGURE 2:**
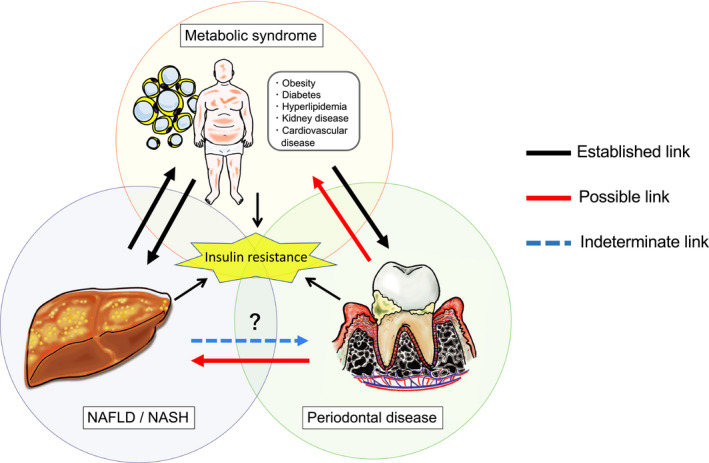
Bidirectional three‐way relationship among metabolic syndrome, nonalcoholic fatty liver disease (NAFLD)/nonalcoholic steatohepatitis (NASH), and periodontal disease, centering on insulin resistance. The black arrows indicate an established link by accumulated evidence. Red arrows indicate possible link that still has unproven causality. Blue arrow indicates indeterminate link because of no or little evidence

Metabolic syndrome is a critical risk factor for both periodontal disease and nonalcoholic fatty liver disease. These diseases mediate a bidirectional three‐way relationship, centered on insulin resistance associated with obesity and diabetes (Figure 2).

### 4.1 Role of metabolic syndrome and insulin resistance in the pathophysiology of nonalcoholic fatty liver disease/nonalcoholic steatohepatitis

Nonalcoholic fatty liver disease is considered the hepatic manifestation of metabolic syndrome because it is closely associated with obesity, insulin resistance, hypertension, and dyslipidemia.^9^, ^10^ Metabolic syndrome is a cluster of metabolic abnormalities that identify individuals who are at risk for diabetes or cardiovascular disease and who are often obese. The diagnostic criteria are defined as the presence of any three of the following five conditions: increased fasting plasma glucose or type 2 diabetes, hypertriglyceridemia, low high‐density lipoprotein cholesterol, hypertension, or increased waist circumference (ethnicity and sex dependence).^103^

It is important to highlight that obesity characterized by excess adipose tissue due to an increase in the number and volume of adipocytes is strongly associated with the development of nonalcoholic fatty liver disease, since it causes fat accumulation in the liver through insulin resistance. Adipose tissue is a multifunctional organ that regulates energy consumption, insulin sensitivity, and inflammatory processes via various inflammatory mediators.^104^, ^105^ In obese people, excess adipose tissue mediates several negative effects, such as increased macrophage infiltration, disruption of adipocytokine production (IL‐1β, IL‐6, tumor necrosis factor alpha, leptin, resistin, visfatin, adiponectin, plasminogen activator inhibitor‐1, etc), and subsequent defective insulin secretion.^106^ Hyperinsulinemia promotes further obesity because insulin is an anabolic hormone that promotes glucose uptake and fat storage. In addition, increased blood levels of proinflammatory adipokines produced in inflamed adipose tissue cause insulin resistance, which is accompanied by low‐grade systemic inflammation, resulting in increased hepatic influx and accumulation of fatty acids.^107^, ^108^, ^109^, ^110^ However, reduction of serum adiponectin, which has an anti‐inflammatory effect, may also induce hepatic fat accumulation, inflammation, and insulin resistance.^106^, ^111^ Many studies have shown that the inflammation occurs as a consequence of obesity, and it may cause insulin resistance and other disturbances of energy homeostasis.

In fact, both excessive body mass index and visceral obesity are recognized as risk factors for nonalcoholic fatty liver disease, and nearly two‐thirds of patients with obesity and type 2 diabetes have hepatic steatosis.^112^, ^113^ In patients with nonalcoholic fatty liver disease, the presence of multiple components of metabolic syndrome are associated with more severe liver disease and are more likely to progress to nonalcoholic steatohepatitis and cirrhosis.^114^, ^115^ Civera et al^116^ reported that obese patients with a higher degree of insulin resistance exhibit more hepatocyte apoptosis in liver biopsy specimens, and this is thought to be mediated by inflammatory cytokines. Furthermore, the presence of metabolic syndrome among nonalcoholic fatty liver disease patients is associated with an increased risk for fibrosis in nonalcoholic steatohepatitis and the risk for eventual liver failure.^117^, ^118^

The liver is not simply a passive participant, since hepatic steatosis has systemic consequences as it worsens metabolic syndrome.^119^ For example, nonalcoholic fatty liver disease itself has been reported to enhance insulin resistance, predict the emergence of metabolic complications, and increase the risk for cardiovascular events.^73^, ^120^ Intracellular lipid content in the liver also decreases insulin clearance, causing the hyperinsulinemia, which is a sign of prediabetes.^121^ In other words, even if nonalcoholic steatohepatitis does not directly lead to end‐stage liver disease, it may have a significant impact by promoting extrahepatic complications in individuals with metabolic syndrome.^122^

### 4.2 Bidirectional relationship between periodontal disease and nonalcoholic fatty liver disease

Given the close connection between nonalcoholic fatty liver disease and metabolic syndrome and the fact that periodontal disease is bidirectionally associated with metabolic syndrome, it is important to consider periodontal disease in the pathology of nonalcoholic fatty liver disease.^55^ Numerous epidemiologic studies have shown that periodontal disease can exacerbate various metabolic disorders, such as diabetes, obesity, dyslipidemia, and chronic kidney disease.^123^, ^124^, ^125^ Periodontitis‐related systemic inflammation may contribute to insulin resistance through elevated blood levels of adipocytokines, such as tumor necrosis factor alpha, IL‐6, and leptin, which inhibit the insulin receptor and its downstream signaling.^126^, ^127^, ^128^, ^129^ The presence of both obesity and periodontal disease significantly increases the risk for diabetes because of the exacerbated insulin resistance due to periodontitis, which further increases glucose and insulin levels in blood.^130^ Insulin resistance also promotes dyslipidemia through increased circulating free fatty acids in blood. Furthermore, periodontal disease is directly and indirectly involved in cardiovascular disease owing to its exacerbation of systemic inflammation and metabolic syndrome.^131^, ^132^ Proinflammatory mediators and periodontopathic bacteria and their products may damage endothelial cells and promote atherogenesis and thrombus formation, thereby increasing the risk for cardiovascular disease.^133^, ^134^, ^135^ Further, intervention studies have reported that periodontal treatment improves insulin resistance, blood glucose levels, lipid profiles, and endothelial function in patients with periodontitis.^136^, ^137^, ^138^, ^139^, ^140^

It is well known that diabetes and obesity negatively impact the progression of periodontal disease.^141^, ^142^ Poor glycemic control in diabetic patients has been correlated with increased risk for periodontal attachment loss and tooth loss compared with nondiabetic subjects.^143^, ^144^ Through the formation of advanced glycation end products and a glucose‐rich environment, diabetes can accelerate the inflammatory process and inhibit wound healing in the periodontal tissues, thereby promoting tissue destruction by periodontitis.^145^ Therefore, the latest classification of periodontal disease includes diabetes as a critical element in determining the grade of periodontitis, and its importance as a risk factor for the progression of periodontal disease is emphasized.^146^ In terms of obese patients, they have approximately twice the risk for periodontal disease and their condition may negatively affect the responsiveness to periodontal treatment compared with normal weight subjects.^129^, ^147^, ^148^ The mechanism by which obesity exacerbates periodontitis is still unclear, but increased adipokines in the gingival crevicular fluid, decreased periodontal immune response, and impaired gingival microcirculation have been proposed.^145^

### 4.3 Association between periodontal disease and nonalcoholic fatty liver disease/nonalcoholic steatohepatitis with a focus on metabolic syndrome

As noted for the various metabolic disorders mentioned previously, periodontal disease can affect nonalcoholic fatty liver disease and nonalcoholic steatohepatitis via disturbances in energy homeostasis. An updated meta‐analysis using four cross‐sectional studies and one retrospective cohort study showed that the association between periodontitis and nonalcoholic fatty liver disease was no longer significant when adjusting for insulin resistance and various metabolic parameters, suggesting that those metabolic conditions (and not periodontitis itself) are predisposing factors for nonalcoholic fatty liver disease.^28^

However, animal studies have shown that periodontal inflammation and infection by periodontal pathogens can cause mild fatty liver and hepatitis, even in healthy animals without metabolic disease.^24^, ^149^ For example, studies using a ligature‐induced periodontitis rodent model have reported an altered hepatic glycolipid metabolism through increased blood levels of inflammatory cytokines, total cholesterol, triglycerides, and oxidative stress.^50^, ^51^, ^149^, ^150^ These metabolic changes in the liver increased the number and size of lipid droplets in hepatocytes, accompanied by hypertrophy of mitochondria and structural changes in the rough endoplasmic reticulum.^59^ Also, oral administration of periodontopathic bacteria, such as *P. gingivalis* and *A. actinomycetemcomitans*, in mice altered the intestinal microbiota and barrier function and caused lipid droplet formation in liver tissues via upregulation of genes related to adipokines, fatty acid biosynthesis, and glucose metabolism.^24^, ^38^ The mice infected with periodontopathic bacteria exhibited impaired glucose tolerance and insulin resistance and showed a slight increase in hepatic fat deposits and inflammation.

Moreover, in animal models of metabolic diseases showing obesity or diabetes, periodontal inflammation and bacterial infection enhanced metabolic disorders in the liver, resulting in accelerated progression of nonalcoholic fatty liver disease.^23^, ^55^, ^151^, ^152^, ^153^, ^154^ Although not all mechanisms explaining the interaction between periodontal disease and metabolic diseases are known, diffusion of inflammatory mediators and reactive oxygen species from inflamed periodontal tissues into the circulation can mediate low‐grade systemic inflammation, and thereby exacerbate insulin resistance in obesity and diabetes.^155^ Ishikawa et al reported that hyperglycemia promotes translocation of *P. gingivalis* from the oral cavity to the liver and reduces hepatic insulin‐induced glycogen biosynthesis in mice.^23^ This fat‐enriched diet‐induced insulin resistance may also be affected by adaptive immunity against *P*. *gingivalis* and its lipopolysaccharide, both through activation of the cervical lymph nodes and the systemic immune response.^153^ A link between periodontitis and insulin resistance has also been demonstrated in adults without diabetes,^156^ overall suggesting that periodontitis may be involved in nonalcoholic fatty liver disease from onset to progression via interactions with metabolic syndrome.

Effects in the opposite direction, namely the effect of liver disease on the periodontal condition, have been presented by a few cross‐sectional studies. Alanine aminotransferase is a liver enzyme commonly used as a surrogate marker for hepatocellular injury, and it has also been proposed as a potential risk indicator for periodontal disease.^29^, ^30^ Furuta et al^30^ found that an elevated serum alanine aminotransferase level was significantly associated with the prevalence of probing pocket depths of 4 mm or more in young Japanese males who presumably had no alcohol consumption habits. Furthermore, Ahmad et al^32^ demonstrated that coexistence of both metabolic syndrome and elevated serum alanine aminotransferase was positively correlated with pocket depth in adult males with low alcohol consumption, but no such association was found in females or males with high alcohol consumption. As already mentioned, nonalcoholic fatty liver disease itself exacerbates metabolic syndrome through enhanced insulin resistance. Components of metabolic syndrome, such as obesity and diabetes, are a significant risk for periodontal disease, and nonalcoholic fatty liver disease may therefore be indirectly involved in the pathophysiology of periodontal disease. However, these studies, because of their cross‐sectional nature, do not support a causal relationship, and the mechanisms involved have not been fully examined. Thus, to the best of our knowledge, there is currently limited evidence that liver disease, at least nonalcoholic fatty liver disease and nonalcoholic steatohepatitis, affects periodontal disease.

## 5 POTENTIAL DUAL PATHWAYS LINKING PERIODONTAL DISEASE AND NONALCOHOLIC FATTY LIVER DISEASE/NONALCOHOLIC STEATOHEPATITIS

**FIGURE 3:**
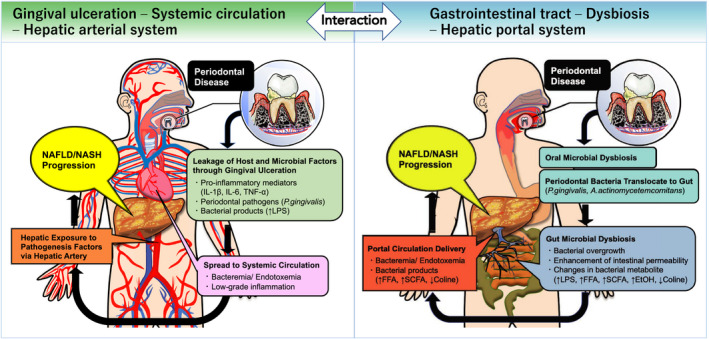
Dual possible pathway for the link between periodontal disease and nonalcoholic fatty liver disease (NAFLD)/nonalcoholic steatohepatitis (NASH). A, One possible mechanism is hematogenous systemic diffusion of bacteria, endotoxin, and inflammatory mediators through microulceration in the periodontal pocket. A proper hepatic artery, which is branched from the abdominal aorta, is presumed the main transportation route from systemic circulation to liver. B, Another mechanism is gut microbial dysbiosis induced by the transport of oral bacteria through the gastrointestinal tract. The oral bacteria–mediated gut dysbiosis can cause impairment of the gut barrier function and immune modulation, further leading to hepatic exposure to bacteremia, endotoxemia, and bacterial metabolite through the enterohepatic circulation by portal vein system. IL‐1β, interleukin 1 beta; IL‐6, interleukin 6; TNF‐α, tumor necrosis factor alpha; FFA, free fatty acids; SCFA; short‐chain fatty acids; EtOH, ethanol

Although the mechanism by which harmful factors are transported from diseased periodontal tissue to the liver is unclear, the following two routes have been proposed based on the unique anatomical characteristics of the liver (Figure 3).

### 5.1 Periodontal microulceration, general circulation, and hepatic arterial system

One possible route connecting periodontal disease and nonalcoholic fatty liver disease/nonalcoholic steatohepatitis is the hematogenous physical diffusion of immunogenic factors and oral pathogenic bacteria from the periodontal tissues. The mechanism linking periodontal disease to systemic disease has long been explained by the concept of microulceration in the periodontal pocket.^1^, ^132^, ^157^, ^158^ The gingival epithelium in a healthy periodontium normally covers the connective tissue, including blood and lymph vessels, and acts as a barrier to obstruct noxious biofilm components.^159^, ^160^ However, in diseased tissues, increases in permeability and microulceration of the gingival epithelium readily allow invasion of noxious substances and microorganisms into the circulation via the periodontal tissues.^157^, ^161^ In addition, inflammation‐induced capillary structural changes, vasodilation, and perturbed blood flow may enhance the diffusion of pathogenic factors.^162^, ^163^

The hematogenous diffusion is known to be further enhanced by mechanical perturbation of the gingival tissues. Studies have revealed that oral mechanical injuries caused by daily dental activity (eg, brushing, flossing, chewing), periodontal procedures (eg, scaling and root planing, probing), and other dental procedures (eg, orthodontics, tooth extraction) cause a bacteremia.^164^, ^165^, ^166^ Patients with periodontal disease show a further increase in serum/circulating bacteria and lipopolysaccharide derived from these oral injuries compared with individuals with healthy periodontal tissue.^167^, ^168^ Specific periodontal pathogens and other oral bacteria have been detected in areas distant from the oral cavity, including atherosclerotic plaques, joint cavities, the brain, and the liver, suggesting their association with various systemic diseases.^22^, ^134^, ^169^, ^170^ Furthermore, periodontal host cells activated by immune interactions with biofilm bacteria enhance the release of reactive oxygen species and inflammatory cytokines, such as IL‐1β, IL‐6, and tumor necrosis factor alpha.^171^, ^172^, ^173^ It has been reported that these pro‐inflammatory cytokines and oxidative stress molecules are elevated in patients with periodontitis, not only in gingival crevicular fluid and gingival tissue but also in serum.^128^, ^129^, ^174^

Therefore, potential liver damage derived from periodontal disease may be delivered to the liver in a hematogenous manner and it may promote the progression of nonalcoholic fatty liver disease and nonalcoholic steatohepatitis. The various substances transferred into the blood via the capillaries of the periodontal tissue first pass through the left and right jugular veins, then they join the superior vena cava and then flow into the heart. After entering the pulmonary circulation for gas exchange, they are pumped from the heart through the aorta and then diffuse throughout the body by the systemic circulation. Regarding the liver, the proper hepatic artery, the potentially vegetative blood vessel of the liver branching from the abdominal aorta, can be presumed the main transportation route.

Indeed, epidemiologic studies have shown that C‐reactive protein, which is synthesized in hepatocytes and activated by proinflammatory cytokines, including tumor necrosis factor alpha and IL‐6, is a modifying factor of periodontitis and nonalcoholic fatty liver disease.^35^, ^40^ Serum C‐reactive protein levels are also known to increase with the severity of periodontal disease. In addition, animal studies have reported that increased serum levels of proinflammatory cytokines and oxidative stress markers, as well as C‐reactive protein, may contribute to nonalcoholic fatty liver disease progression after inducing periodontitis.^20^, ^149^, ^151^ Our previous study also showed that adding *P. gingivalis* to ligature‐induced periodontitis in rats exacerbated nonalcoholic fatty liver disease, which was accompanied by increased serum lipopolysaccharide activity and C‐reactive protein.^53^ These data suggest that periodontally derived circulating inflammatory molecules play a critical role in the pathogenesis of nonalcoholic fatty liver disease and nonalcoholic steatohepatitis.

As for periodontopathic bacteria, Furusho et al^22^ reported that *P. gingivalis* was detected by immunochemical staining in 52.5% of liver biopsy specimens from nonalcoholic steatohepatitis patients. The *P*. *gingivalis*–positive liver cases showed a significantly higher fibrosis score than the *P*. *gingivalis*–negative cases. Furthermore, Ishikawa et al^23^ found that orally administered SNAP26b‐tagged *P. gingivalis* in mice was detected in the liver tissue, and the translocation of *P. gingivalis* from the oral cavity to the liver was further promoted by hyperglycemia. Interestingly, *P. gingivalis* is known to have the ability to invade and survive inside immune cells, such as macrophages and dendritic cells,^175^, ^176^ suggesting that periodontopathic bacteria may hijack circulating leukocytes to serve as Trojan horses for dissemination of infection from the oral cavity to the liver.^177^

### 5.2 Gut microbial dysbiosis and enterohepatic circulation

Another potential route of communication between the oral cavity and the liver is via transport of oral bacteria through the gastrointestinal tract. A person swallows up to 1.5 L of saliva, which would equate to 1.5 × 10^12^ oral bacteria per day.^178^, ^179^, ^180^, ^181^ Schmidt et al^182^ reported that, despite the harsh acidic gastric environment, the presence of oral microbes in the gut is common even among healthy individuals. This indicates that the oral microbiota may be contributors to the intestinal microbiome. The resident gut bacteria are generally considered to be the major barrier preventing ectopic colonization by swallowed oral bacteria. However, disruption of healthy gut microbiota can promote intestinal colonization by oral bacteria.^183^, ^184^ For instance, multiple factors, such as use of antibiotics, enteritis, diet, drinking habits, and obesity, may promote opportunistic gut colonization by oral bacteria that may mediate gut dysbiosis. Lourenço et al^185^ showed that numerous oral taxa related to periodontal destruction and inflammation were detected in the gut microbiota of individuals regardless of periodontal status. However, patients with periodontal disease had a less diverse gut microbiota characterized by an increased ratio of Firmicutes‐Bacteroidetes and enrichment in Euryarcheota, Verrucomicrobiota, and Proteobacteria compared with individuals with a healthy periodontal condition.

It is widely known that gut microbiome dysbiosis is closely associated with nonalcoholic fatty liver disease and nonalcoholic steatohepatitis.^186^, ^187^, ^188^ All blood from the gut travels via the portal vein to reach the liver, which performs the metabolic, immunological, and detoxification processes before the blood reaches the systemic circulation.^5^, ^186^ Therefore, through the enterohepatic circulation, the liver is constantly exposed to bacterial components and metabolites absorbed from the gut, which can potentially affect the condition of the liver. In fact, it is known that in gut dysbiosis there is an increase in choline metabolism (which is essential for lipolysis), hepatotoxins (such as lipopolysaccharide and ethanol), and volatile organic compounds.^189^, ^190^, ^191^, ^192^ Furthermore, dysbiosis enhances intestinal permeability by impairing intercellular tight junctions in the gut wall and promotes the transfer of hepatotoxins and enterobacteria to the liver.^193^, ^194^

From the foregoing, it has been suggested that dysbiosis due to the intestinal translocation of oral bacteria may be involved in the pathogenesis of nonalcoholic fatty liver disease. Several animal studies have demonstrated that oral administration of periodontopathic bacteria, including *P. gingivalis* and *A. actinomycetemcomitans*, was associated with changes in gut microbiota, as well as in glucose and lipid metabolic pathways, leading to insulin resistance and hepatic fat deposition.^24^, ^38^ *P. gingivalis*–induced gut dysbiosis further downregulated gene expression of tight junction proteins that participate in gut barrier function and increased serum lipopolysaccharide levels.^24^, ^25^ In contrast, Blasco‐Baque et al^153^ found that mice, fed a high‐fat diet and orally inoculated with *P. gingivalis*, *F. nucleatum*, and *Prevotella intermedia*, exhibited impaired glycemic metabolism and insulin resistance without remarkable changes in their gut microbiome. Similarly, Ohtsu et al^58^ reported that, in streptozotocin‐induced diabetic mice, *P. gingivalis* increased the expression of inflammatory genes, such as tumor necrosis factor alpha and C‐C motif chemokine ligand 2, in the liver but caused only small modifications in the gut microbiota without suppression of tight junction proteins.

Taken together, the mechanism by which oral bacteria induce gut dysbiosis that contributes to nonalcoholic fatty liver disease pathology is presently unclear, because of inconsistent results in different animal models. In addition, there have been no studies in humans showing a relationship between periodontitis‐associated gut dysbiosis and nonalcoholic fatty liver disease. To clarify the clinical relevance of periodontal disease in the progression of nonalcoholic fatty liver disease via gut microbiota, more studies are needed, including strictly controlled animal studies and large‐scale epidemiologic studies.

## 6 POTENTIAL MECHANISMS BY WHICH PERIODONTAL DISEASE MAY INCREASE THE RISK OF NONALCOHOLIC FATTY LIVER DISEASE/NONALCOHOLIC STEATOHEPATITIS

**FIGURE 4:**
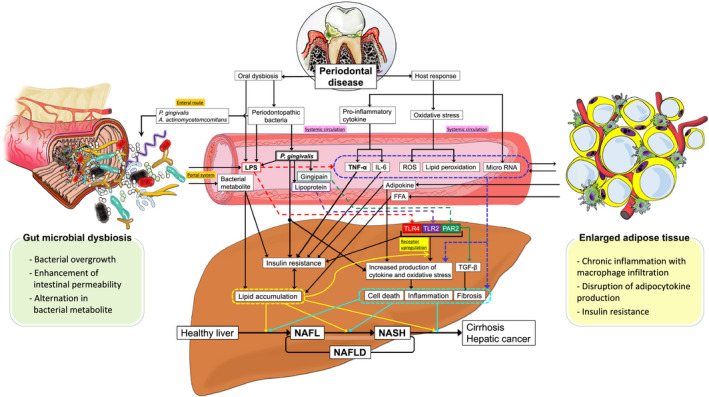
Mechanisms through which periodontal disease increases the risk of nonalcoholic fatty liver disease (NAFLD)/nonalcoholic steatohepatitis (NASH). LPS, lipopolysaccharide; TNF‐α, tumor necrosis factor alpha; IL‐6, interleukin 6; ROS, reactive oxygen species; FFA, free fatty acids; TLR, toll‐like receptor; PAR2, protease activated receptor 2; TGF‐β, transforming growth factor beta

The liver, which is located at a hemodynamic convergence point in the body, connects the hepatic arterial and portal systems, allowing a mixture of oxygenated blood and blood from the portal system. Therefore, in a state of periodontitis, the liver is under constant exposure to various pathogenic factors that are diffused systemically from the oral cavity, such as bacteria and their components, inflammatory cytokines, and reactive oxygen species, and these can be involved in the disease promotion of nonalcoholic fatty liver disease and nonalcoholic steatohepatitis (Figure 4).

### 6.1 Periodontopathic bacteria

Data from over the last decade strongly suggest that *P. gingivalis* is involved in nonalcoholic fatty liver disease and nonalcoholic steatohepatitis. *P. gingivalis* has many virulence factors (such as collagenase, aminopeptidase, and a trypsin‐like enzyme) and other components (including lipopolysaccharide and fimbriae) that are also known to trigger intracellular signaling events.^195^

Yoneda et al,^21^ using polymerase chain reaction assays, analyzed various periodontopathic bacteria in saliva collected from nonalcoholic fatty liver disease patients and non‐nonalcoholic fatty liver disease control subjects and found that the detection frequency of *P. gingivalis* was significantly higher in the nonalcoholic fatty liver disease patients than in the non‐nonalcoholic fatty liver disease subjects. Fifty percent of *P. gingivalis* fimbriae detected in the nonalcoholic fatty liver disease patients were type II, which are known to be part of invasive genotypes. In addition, in a nonalcoholic fatty liver disease mouse model fed a high‐fat diet, administration of type II *P. gingivalis* via the jugular vein dramatically accelerated nonalcoholic fatty liver disease progression. In contrast, a cross‐sectional study by Nakahara et al^42^ reported that advanced liver fibrosis was significantly correlated with serum immunoglobulin G antibody titers against *P*. *gingivalis* fimbriae type IV but not type II in liver biopsy–proven nonalcoholic fatty liver disease patients. The authors also showed that infection with type IV *P. gingivalis* via the pulp cavity promoted hepatic fatty acid metabolism and fibrosis in a nonalcoholic fatty liver disease mouse model fed a high‐fat diet. These differences in the impact of different *P. gingivalis* fimbriae types on risk for nonalcoholic fatty liver disease may depend on multiple factors, including the sample type, the analysis method, the severity of nonalcoholic fatty liver disease, and differences in/unreported periodontal parameters. Furthermore, the results from these animal models should be interpreted with caution because *P. gingivalis* was directly administered via the tail vein or pulp cavity rather than via the periodontal tissue.

Our previous animal studies showed that a combination of *P. gingivalis* infection with ligature‐induced periodontitis increased serum levels of alanine aminotransferase and lipopolysaccharide as well as hepatic fat deposition in rats with high‐fat diet–induced obesity and insulin resistance.^53^, ^55^ However, the intervention with either *P. gingivalis* or ligature placement alone did not show similar changes. Therefore, our results suggest that *P. gingivalis* or its products may enter the blood circulation via the inflamed periodontal tissues and thereby contribute to nonalcoholic steatohepatitis progression. As already mentioned (Section 5.1), it is known that *P. gingivalis* can diffuse from the oral cavity to the systemic circulation and reach the liver.^22^, ^23^

Some studies have clarified the molecular mechanism and explained the direct effect of *P. gingivalis* on liver tissue. For example, Ishikawa et al^23^ reported that *P. gingivalis* was internalized into human hepatocyte HepG2 cells and thereby suppressed glycogen synthesis by attenuating the insulin‐induced phosphorylation of insulin receptor substrate 1, serine/threonine kinase Akt, and glycogen synthase kinase 3 beta. *P. gingivalis* also decreased the insulin‐induced phosphorylation of Forkhead box protein O1, which is a transcription factor regulating hepatic gluconeogenesis, and attenuated the Forkhead box protein O1 translocation to hepatocytes.^196^ In addition, a *P*. *gingivalis* trypsin‐like gingipain enzyme is translocated to mouse liver with the outer membrane vesicles of *P*. *gingivalis* and it suppresses Akt/glycogen synthase kinase 3 beta signaling, resulting in attenuation of hepatic glycogen synthesis with hyperglycemia in response to insulin.^197^ Using an in vitro model of nonalcoholic fatty liver disease that is mediated by treating HepG2 cells with oleic acid, Zaitsu et al^198^ found that intracellular lipid droplets suppress the elimination of *P. gingivalis* from hepatic cells by altering lysosome formation and autophagy at an early phase of infection.

In vivo and in vitro studies by Nagasaki et al^52^ showed that *P*. *gingivalis* infection activated hepatic stellate cells via transforming growth factor beta 1/Smad and /extracellular signal–regulated kinases signaling pathways and was associated with liver fibrosis. Specifically, *P. gingivalis* gingipain induces transforming growth factor beta 1 production via proteinase‐activated receptor 2, which then upregulates phosphorylation of Smad and extracellular signal–regulated kinases via the transforming growth factor beta 1 receptor I/II complex, subsequently resulting in hepatic stellate cells activation in an autocrine manner. In addition, the *P. gingivalis* lipoprotein induces galectin‐3 production by hepatic stellate cells via toll‐like receptor 2 signal transduction and it stabilizes transforming growth factor beta receptor II to increase sensitivity to transforming growth factor beta 1. Transforming growth factor beta 1 and galectin‐3 produced from steatotic hepatocytes following *P. gingivalis* infection also contribute to the enhancement of hepatic stellate cells activation in a paracrine manner. These pathways may be further accelerated in fatty liver because expression of proteinase‐activated receptor 2 and toll‐like receptor 2 is significantly upregulated by hepatic fat accumulation.

In contrast, other studies have proposed a mechanism in which swallowed periodontopathic bacteria, including *P. gingivalis* and *A. actinomycetemcomitans*, induce gut dysbiosis, explaining an indirect relationship between periodontal disease and nonalcoholic fatty liver disease (see Section 5.2, Figure 3). However, little is known about the mechanism by which stimulation by single periodontopathic bacteria is associated with changes in the gut microbiota, its bacterial metabolites, and subsequent host responses. According to a recent report by Kitamoto et al,^184^ periodontitis promotes the growth of even indigenous oral bacteria, such as *Klebsiella* and *Enterobacter* species, which do not show remarkable pathogenicity in the oral cavity, yet ectopic gut colonization by these bacteria may play an important role in worsening gut inflammation. Therefore, it would be desirable to comprehensively evaluate the relationship between periodontitis‐induced changes in the entire oral microbiota, gut dysbiosis, and nonalcoholic fatty liver disease, rather than evaluating relationships with just individual periodontopathic bacteria.

### 6.2 Lipopolysaccharide and endotoxemia

Lipopolysaccharide (endotoxin) is a major component of the cell wall of gram‐negative bacteria and is normally released extracellularly following the destruction and degradation of bacteria.^199^ The active center of lipopolysaccharide is held by lipid A, which exerts harmful effects on humans and animals, such as pyrogenicity, proinflammatory responses, and lethal toxicity. Most cells of the innate immune system express lipopolysaccharide receptors, which consist of pattern recognition receptors, such as toll‐like receptor 4 and CD14, and these initiate a powerful inflammatory cascade in response to lipopolysaccharide.^200^

In general, the enhancement of gut‐derived endotoxemia is caused by small intestinal bacterial overgrowth with dysbiosis and increased intestinal permeability, which is considered to be critically involved in the onset and progression of nonalcoholic fatty liver disease. In fact, patients with nonalcoholic steatohepatitis have elevated blood lipopolysaccharide levels compared with individuals with healthy livers.^193^, ^194^, ^201^ Moreover, the inhibition of lipopolysaccharide receptors was associated with significant protection against the development of nonalcoholic fatty liver disease in various animal models.^202^, ^203^

In patients with periodontal disease, the degree and frequency of endotoxemia is increased with the severity of the disease,^168^, ^204^ which contributes to systemic inflammation, including increased blood levels of C‐reactive protein, IL‐6, and tumor necrosis factor alpha.^205^, ^206^ During the development of oral dysbiosis, periodontopathic bacteria of the genera *Fusobacterium*, *Porphyromonas*, and *Prevotella* are predominant in the periodontal microenvironment, thereby promoting lipopolysaccharide production.^207^ The endotoxemia can be explained not only by the translocation of lipopolysaccharide from inflamed periodontal tissue to the systemic circulation, but also by liver exposure to lipopolysaccharide via the portal system due to periodontitis‐induced gut dysbiosis (see Section 5, Figure 3).

Although a trace amount of lipopolysaccharide (about 1.0 ng/mL) is usually present in the portal circulation even under normal physiologic conditions, the liver of a healthy subject is hardly responsive to such low concentrations of this endotoxin.^208^ In this regard, it is known that fatty liver increases hepatic macrophages (Kupffer cells) and enhances their susceptibility to the low‐dose lipopolysaccharide.^209^ Imajo et al^210^ revealed that, in mouse fatty liver induced by a high‐fat diet, the sensitivity to low‐dose lipopolysaccharide was enhanced by an upregulation CD14 expression via the reptin/signal transducer and activator of transcription 3 signaling in Kupffer cells. Thus, endotoxemia (even low‐dose lipopolysaccharide) derived from periodontal disease is likely to be involved in the progression to nonalcoholic steatohepatitis, including inflammation and fibrosis of the fatty liver of patients with metabolic disease, such as obesity and diabetes.

Interestingly, a fatty liver upregulates not only toll‐like receptor 4 but also toll‐like receptor 2, and lipopolysaccharide of *P. gingivalis* activates hepatocyte inflammasomes (nucleotide oligomerization domain‐like receptor family pyrin domain containing 3 and caspase‐1) and proinflammatory cytokines via toll‐like receptor 2–dependent pathways.^22^ Thus, a fatty liver promotes responsiveness to *P. gingivalis*. Although the toll‐like receptor 4/myeloid differentiation factor 2–dependent biological activity of *P. gingivalis* lipopolysaccharide is known to be significantly weaker than that of *Escherichia coli* or *Salmonella* species, its lipid A component elicits a strong host immune response via toll‐like receptor 2 rather than toll‐like receptor 4/myeloid differentiation factor 2. However, Ogawa et al^211^ found that the main biological activity of *P. gingivalis* lipopolysaccharide via this toll‐like receptor 2 pathway is derived from a lipoprotein, which is composed of a triacylated *S*‐(2,3‐dihydroxypropyl)cysteine, rather than the lipid A. The lipopolysaccharide fraction extracted from *P. gingivalis* is strongly contaminated with the lipoprotein, and it is extremely difficult to remove the lipoprotein during the process of lipopolysaccharide purification.^212^, ^213^ Indeed, Ding et al^214^ reported that more intracellular lipids accumulated in the oleic acid–treated human hepatocellular cells when stimulated with *P. gingivalis* lipopolysaccharide compared with *E. coli* lipopolysaccharide. Sasaki et al^56^ showed that an intravenous injection of sonicated *P. gingivalis*–derived components containing lipopolysaccharide caused insulin resistance, impaired glucose tolerance, led to gut microbial alterations, and increased levels of fatty liver in mice fed a high‐fat diet.

Our research team administered *P*. *gingivalis*–derived lipopolysaccharide double‐labeled with hydrogen‐3 and carbon‐14 into the palatal gingiva of normal or diet‐induced obese rats to clarify the pharmacokinetics of *P*. *gingivalis* lipopolysaccharide in vivo over time.^54^ The results showed that most of the lipopolysaccharide spread through the circulation and accumulated markedly in the liver more than in other organs, including the kidney, brain, and spleen. It is noteworthy that this accumulation of *P. gingivalis* lipopolysaccharide was increased and maintained in the fatty liver for a longer period of time than in the healthy liver. Furthermore, in ongoing studies in our laboratory, we are finding that the high‐fat diet may delay the metabolic clearance of *P. gingivalis* lipopolysaccharide from the liver. This change in lipopolysaccharide kinetics in fatty liver may be due to the aforementioned increased Kupffer cells and upregulation of toll‐like receptor 4 and toll‐like receptor 2 pathways.

### 6.3 Proinflammatory cytokines and adipokines

Adipokines, such as tumor necrosis factor alpha, IL‐6, leptin, and adiponectin produced by adipose tissue, are closely involved with hepatic lipid deposition, inflammation, fibrosis, and carcinogenesis in nonalcoholic fatty liver disease.^215^ In the enlarged adipose tissue of obese people, increased secretion of chemoattractant protein‐1 causes an infiltration of inflammatory cells, primarily macrophages, which then secrete inflammatory cytokines and chemokines that disrupt the balance of adipokine production by adipocytes.^216^ Adipokines affect not only chronic inflammation and insulin resistance in local adipose tissue, but also hepatic insulin sensitivity directly.^81^

Periodontal disease is characterized by a low‐grade systemic inflammatory state that increases blood levels of proinflammatory cytokines, including tumor necrosis factor alpha and IL‐6, similar to obesity, suggesting a potential risk for nonalcoholic fatty liver disease in the bidirectional relationship between periodontal disease and obesity (see Section 4, Figure 2). In particular, tumor necrosis factor alpha plays a major role in hepatic insulin resistance, and it inactivates the insulin receptor substrate by serine phosphorylation through activation of a serine/threonine kinase, thus blocking the insulin receptor signaling cascade.^217^ IL‐6, which is upregulated by tumor necrosis factor alpha, is also associated with decreased insulin signaling and induction of fatty acid oxidation, as well as secretion of C‐reactive protein by the liver.^145^, ^218^

Like adipocytes, cells within periodontal tissue can also secrete various adipokines.^145^, ^219^, ^221^ Patients with periodontitis have elevated serum levels of proinflammatory adipocytokines, such as leptin, visfatin, and resistin, and reduced serum levels of the anti‐inflammatory adipokine adiponectin.^128^, ^222^, ^223^, ^224^ Recent studies have shown that leptin normally plays a central role in suppressing lipid accumulation in the liver both by anorectic action and improving glycolipid metabolism, although it is also involved in liver fibrosis and hepatocellular carcinoma formation.^225^, ^226^ On the other hand, adiponectin, which is a beneficial adipokine, promotes hepatic fatty acid metabolism by activating adenosine monophosphate–activated protein kinase and peroxisome proliferator–activated receptor α, in addition to enhancing insulin sensitivity and anti‐inflammatory actions.^218^ For this reason, clinical studies have shown that hypoadiponectinemia is a risk factor for the development of metabolic syndrome and nonalcoholic steatohepatitis.^227^, ^228^, ^229^

### 6.4 Oxidative stress

Oxidative stress is defined as a deleterious condition resulting from an imbalance between reactive oxygen species and antioxidant capacity.^230^, ^231^ Reactive oxygen species is a collective term that broadly describes a variety of molecules derived from oxygen molecules and free radicals: singlet oxygen, superoxide, hydrogen peroxide, hydroxyl, and nitric oxide.^232^ Under physiologic conditions, these reactive oxygen species effects are rapidly eliminated by antioxidant defenses and repair enzymes in the body.^233^ However, when excessive reactive oxygen species are produced, this causes nonspecific cell death and tissue injury through oxidative damage to deoxyribonucleic acid (DNA), fatty acids, and proteins due to their high reactivity.

In the development of periodontal disease, activated polymorphonuclear leukocytes produce a large amount of reactive oxygen species, which are involved in periodontal tissue destruction.^234^, ^235^ Oxidative stress is also one of the major mediators used to explain the mechanism connecting periodontitis and systemic diseases, because it is associated with various diseases, including periodontitis, obesity, and nonalcoholic fatty liver disease.^236^ Indeed, some studies have shown evidence that periodontal inflammation may be involved in systemic oxidative stress. In a meta‐analysis that included 16 studies from different countries, Liu et al^236^ showed that serum levels of total antioxidant capacity were lower and levels of nitric oxide and malondialdehyde were higher in patients with chronic periodontitis than in healthy subjects. Nitric oxide is a short‐lived reactive free radical, and malondialdehyde is a major product of polyunsaturated fatty acid peroxidation useful for assessing increased oxidative stress.^237^ Furthermore, clinical intervention with periodontal therapy improved elevated serum levels of reactive oxygen species and lipid peroxides in patients with periodontitis.^238^, ^239^ These results suggest that the hematogenous diffusion of periodontitis‐derived reactive oxygen species and oxidative products induce systemic oxidative stress. In addition, activation of polymorphonuclear leukocytes in peripheral blood may also increase the circulating reactive oxygen species. Matthews et al^240^ found that peripheral neutrophils collected from chronic periodontitis patients show increased production and release of reactive oxygen species in vitro. Dias et al^241^ reported that increased inflammatory cytokines (including IL‐8, interferon gamma, and granulocyte‐macrophage colony–stimulating factor) in plasma from periodontitis patients were significantly more effective in directly stimulating neutrophil superoxide production compared with those in healthy subjects.

Therefore, periodontitis‐related systemic oxidative stress may be involved in the oxidative damage to the liver. A series of animal studies by Tomofuji and coworkers^19^, ^20^, ^242^ revealed that elevated blood reactive oxygen species and lipid peroxide hexanoyl‐lysine following periodontal inflammation were involved in oxidative DNA damage and apoptosis in the liver of rats. Other studies also reported that ligature‐induced periodontitis in rats induced mild hepatic damage through increased malondialdehyde and decreased antioxidant glutathione production present in both the blood and liver.^50^, ^51^, ^57^, ^150^ Furthermore, a high‐fat or high cholesterol diet cooperatively with periodontitis enhanced intrahepatic oxidative stress, resulting in exacerbation of steatohepatitis.^152^, ^154^

### 6.5 **Micro**–**ribonucleic acid**

Micro–ribonucleic acids (RNAs), which are endogenous noncoding regulatory RNAs, have important functions in posttranscriptional gene regulation. MicroRNAs may be a new potential factor linking periodontal disease and liver disease. MicroRNAs can bind complementary sequences in untranslated regions of various target messenger RNAs, leading to degradation or translational repression of messenger RNAs, which can contribute to a wide range of biological activities, such as cell differentiation, organogenesis, inflammatory responses, and carcinogenesis.^243^, ^244^ MicroRNAs may also play an important role in the pathogenesis of nonalcoholic fatty liver disease, and they have recently been explored as new molecular markers for the diagnosis and prognosis of fatty liver.^245^ In addition, circulating microRNAs from some organs, such as adipose tissue, are known to act as metabolic regulators and alter specific gene expression in the liver.^246^ Although the study of microRNAs in periodontology is still at an early stage, one study using a ligature‐induced periodontitis mouse model reported that changes in blood microRNAs were consistent with hepatic apoptosis–related messenger RNA expression.^247^

## 7 IMPACT OF PERIODONTAL THERAPY ON NONALCOHOLIC FATTY LIVER DISEASE PATIENTS

Few intervention studies have examined the effect of periodontal treatment on nonalcoholic fatty liver disease. Yoneda et al^21^ performed periodontal treatment in 10 patients with nonalcoholic fatty liver disease who had periodontitis, which was defined by the presence of periodontal pockets of 5 mm or more in at least four sites. Oral hygiene instruction, scaling and root planing, and local administration of hydrochloric minocycline were performed. Decreased levels of aspartate aminotransferase and alanine aminotransferase were found 1 month after the baseline, and the decrease reached statistical significance after 2 months; after 3 months, a further decline was observed. Bajaj et al^61^ treated 26 cirrhotic and 20 age‐matched noncirrhotic patients with gingivitis and mild or moderate periodontitis with oral hygiene instruction and scaling and root planing. Another 24 cirrhotic patients that did not receive periodontal therapy were followed for the same period of time. Patients with cirrhosis, especially those with hepatic encephalopathy, showed improvements in their dysbiosis in stool and saliva samples, as well as improvements in endotoxin, lipopolysaccharide‐binding protein, and saliva and serum inflammatory mediators after periodontal treatment. In the group of patients with cirrhosis who did not receive periodontal therapy, there was an increase in endotoxin and lipopolysaccharide‐binding protein levels during the same period. However, these studies had limitations, including the lack of a control group, or if a control group was available, it was not randomized, and data on periodontal parameters were not presented. In an upcoming study, Kamata et al^60^ perform a multicenter, randomized controlled trial comparing between the effects of scaling and root planing and/or oral hygiene on serum alanine aminotransferase and immunoglobulin G antibody titer for *P. gingivalis* for 12 weeks.

## 8 ORAL AND GUT MICROBIOME–TARGETED PROBIOTIC THERAPY IN MANAGEMENT OF NONALCOHOLIC FATTY LIVER DISEASE

Periodontal disease is currently considered to be the result of a harmful shift in the balance of the normally stable resident oral microbiota.^248^ As mentioned earlier, gut dysbiosis induced by enteral translocation of periodontopathic bacteria may be involved in nonalcoholic fatty liver disease. One mechanism assumed to link the gut microbiome with nonalcoholic fatty liver disease is the disruption of the gut epithelial barrier, which may allow leakage of microbial products and metabolites into the portal circulation. Namely, changes in lipopolysaccharide and bacterial metabolites due to gut dysbiosis can induce intestinal inflammation and increase permeability, thereby promoting hepatic exposure to these components, which can directly cause nonalcoholic fatty liver disease and liver fibrosis.^249^ Thus, there is increasing interest in the potential of the human oral and gut microbiome to serve as a target for prophylactic and therapeutic interventions in nonalcoholic fatty liver disease.

Diverse strategies for manipulating the gut microbiome in the management of nonalcoholic fatty liver disease have been proposed, including the use of antibiotics, probiotics, prebiotics, and symbiotics (a combination of probiotics and prebiotics). Probiotics are defined as live cultures of microorganisms that are beneficial to the human body.^250^ Prebiotics, fermentable foods that contain dietary fiber, have an indirect effect on the human body by affecting the activity of probiotics.^251^ Antibiotics exert beneficial effects on metabolic disorders by nonspecifically suppressing the microbiome, but they may be accompanied by harmful side effects and potential emergence of antibiotic‐resistant bacterial strains. Therefore, recently, supplementation with probiotics and symbiotics in the treatment of nonalcoholic fatty liver disease has been favorably accepted due potential enhanced safety for humans and the environment.^252^, ^253^, ^254^ Preclinical animal studies have shown that probiotics suppress the development of insulin resistance and hepatic inflammatory signaling and improve steatosis through regulation of the gut microbiota.^255^, ^256^, ^257^, ^258^ A recent systematic meta‐analysis by Sharpton et al,^252^ which consisted of 21 randomized clinical trials, revealed that the use of probiotics or symbiotics improved liver‐specific markers of hepatic function (alanine aminotransferase), liver stiffness measurements, and liver steatosis in patients diagnosed with nonalcoholic fatty liver disease.

In the oral context, the application of probiotics in the treatment of gingivitis and periodontitis can improve microbiological outcomes in saliva and subgingival plaque with or without nonsurgical periodontal treatment, such as scaling and root planing.^259^ Probiotics, whether as monotherapy or as adjunctive agents, also show beneficial effects on periodontal parameters, including plaque index, gingival index, bleeding on probing, clinical attachment levels, gingival crevicular fluid volume, and host response factors, although the magnitudes of clinical changes in some cases were limited compared with the effects on the microbiological outcomes.

Recently, our studies have reported that an antimicrobial peptide, nisin, which is produced primarily by *Lactococcus* species, has effectiveness in the context of periodontal disease.^259^, ^260^ Nisin, a type of bacteriocin, belongs to a group of cationic peptide antimicrobials collectively called Type A (I) lantibiotics.^261^ Nisin and other lantibiotics have gathered a lot of attention in the food industry and the medical field because of their potent and broad‐spectrum activity even at trace concentrations, low cytotoxicity at antibacterial concentrations, and low likelihood of promoting the development of bacterial resistance.^262^, ^263^, ^264^, ^265^ Interestingly, our data showed that in oral salivary–derived biofilms, nisin‐producing *Lactococcus lactis* and nisin reduce the levels of bacterial pathogens while retaining oral commensal bacteria, such as *Neisseria* species.^260^ The probiotic *L. lactis* and nisin also significantly inhibited the formation, structure, and viability of biofilms spiked with periodontopathic bacteria. We further found that oral administration of the probiotic *L. lactis* prevents alveolar bone loss and gingival inflammation in a polymicrobial mouse model of periodontal disease.^259^, ^266^

However, little is known about the significance of probiotics, symbiotics, and bacteriocins for the management of nonalcoholic fatty liver disease in patients with periodontal disease. In an ongoing study in our laboratory, we are exploring the role of nisin in preventing oral polymicrobial infection–induced gut microbiome changes and liver steatosis in mice, and a detailed analysis of specific changes in the microbiome composition and hepatic immune response is still underway.

Jena et al^267^ reported that *Lactococcus* protects the liver from inflammation in mice with western diet–induced gut dysbiosis. Ansari et al^268^ have shown that a fermented herbal formula containing *L. lactis* effectively improved serum liver function markers and hepatic fat deposition. These studies support the significant potential for using the probiotic *L. lactis* and nisin for prophylaxis and treatment of nonalcoholic fatty liver disease in patients with periodontal disease.

Therefore, probiotics and bacteriocins are promising therapeutic strategies to address the complications of periodontal disease and nonalcoholic fatty liver disease. However, the development of oral and gut microbiome–targeted therapy is currently ongoing and is at an early phase of study. Gaining further evidence of microbiome‐targeted therapies in the management of nonalcoholic fatty liver disease will require a further understanding of the effects of probiotics and bacteriocins on host immune regulation, differences in delivery methods, and long‐term changes in microbial composition and functional maintenance.

## 9 CONCLUDING REMARKS

Growing evidence from clinical and basic studies supports the relationship between periodontal disease and nonalcoholic fatty liver disease. Extensive research has established plausible mechanisms to explain how periodontal disease can negatively affect nonalcoholic fatty liver disease and nonalcoholic steatohepatitis. In particular, in a population with components of metabolic syndrome, the interaction between periodontitis and systemic conditions related to insulin resistance further strengthens the association with nonalcoholic fatty liver disease.

However, most of the pathologic links between periodontitis and nonalcoholic fatty liver disease in humans are provided by epidemiologic observational studies, and the causal relationship has not yet been established. Several systematic and meta‐analysis studies show conflicting results. In addition, the effect of periodontal treatment on nonalcoholic fatty liver disease has hardly been studied, as there is only limited evidence available from a single‐arm intervention study.

Even so, given the global burden of periodontal disease combined with the recent nonalcoholic fatty liver disease epidemic, this fact has important clinical and public health implications. In the future, if it becomes possible to clearly distinguish nonalcoholic steatohepatitis that has a definite association with periodontal disease, there may be a specific case definition for “periodontal disease–based chronic liver disease”, or “periodontal disease–related nonalcoholic fatty liver disease (PNAFLD)” or “periodontal disease–related nonalcoholic steatohepatitis (PNASH)”.

To accomplish the goal, further research is needed to elucidate the mechanism by which periodontopathic bacteria, lipopolysaccharide, and proinflammatory mediators translocate to the liver and the precise role of periodontal disease in the pathogenesis of nonalcoholic fatty liver disease. In parallel, further epidemiologic cohort studies and randomized controlled trials are needed to determine the clinical relevance of periodontal disease in the development of nonalcoholic fatty liver disease. These efforts will pave the way for new approaches based on a periodontological viewpoint that will enable early diagnosis and therapeutic intervention of this life‐threatening liver disease.

## CONFLICT OF INTEREST

The authors declare no potential conflicts of interest with respect to the authorship and/or publication of this article.
